# Comprehensive Transcriptome Profiling of Dairy Goat Mammary Gland Identifies Genes and Networks Crucial for Lactation and Fatty Acid Metabolism

**DOI:** 10.3389/fgene.2020.00878

**Published:** 2020-09-25

**Authors:** Cong Li, Jiangjiang Zhu, Hengbo Shi, Jun Luo, Wangsheng Zhao, Huaiping Shi, Huifen Xu, Hui Wang, Juan J. Loor

**Affiliations:** ^1^Key Laboratory of Animal Genetics, Breeding and Reproduction of Shaanxi Province, College of Animal Science and Technology, Northwest A&F University, Xianyang, China; ^2^Key Laboratory of Qinghai-Tibetan Plateau Animal Genetic Resource Reservation and Utilization, Sichuan Province and Ministry of Education, Southwest Minzu University, Chengdu, China; ^3^College of Animal Science, Zhejiang University, Hangzhou, China; ^4^Mammalian NutriPhysioGenomics, Department of Animal Sciences and Division of Nutritional Sciences, University of Illinois, Urbana, IL, United States

**Keywords:** dairy goat, digital gene expression sequencing, lactation, mammary gland, fatty acid metabolism

## Abstract

Milk fatty acids secreted by the mammary gland are one of the most important determinants of the nutritional value of goat milk. Unlike cow milk, limited data are available on the transcriptome-wide changes across stages of lactation in dairy goats. In this study, goat mammary gland tissue collected at peak lactation, cessation of milking, and involution were analyzed with digital gene expression (DGE) sequencing to generate longitudinal transcript profiles. A total of 51,299 unigenes were identified and further annotated to 12,763 genes, of which 9,131 were differentially expressed across various stages of lactation. Most abundant genes and differentially expressed genes (DEGs) were functionally classified through clusters of euKaryotic Orthologous Groups (KOG), Gene Ontology (GO), and Kyoto Encyclopedia of Genes and Genomes (KEGG) databases. A total of 16 possible expression patterns were uncovered, and 13 genes were deemed novel candidates for regulation of lactation in the goat: *POLG*, *SPTA1*, *KLC*, *GIT2*, *COPS3*, *PDP*, *CD31*, *USP16/29/37*, *TLL1*, *NCAPH*, *ABI2*, *DNAJC4*, and *MAPK8IP3*. In addition, *PLA2*, *CPT1*, *PLD*, *GGA*, *SRPRB*, and *AP4S1* are proposed as novel and promising candidates regulating mammary fatty acid metabolism. “Butirosin and neomycin biosynthesis” and “Glyoxylate and dicarboxylate metabolism” were the most impacted pathways, and revealed novel metabolic alterations in lipid metabolism as lactation progressed. Overall, the present study provides new insights into the synthesis and metabolism of fatty acids and lipid species in the mammary gland along with more detailed information on molecular regulation of lactogenesis. The major findings will benefit efforts to further improve milk quality in dairy goats.

## Introduction

Goat milk contains many macro- and micro-nutrients that are essential for the growth and development of a newborn, and has been recognized as beneficial for human health (German et al., 2002; Casado et al., 2009). The nutritional value of goat milk is mainly attributable to its fat and protein fractions, which are critically important to provide both energy and essential nutrients for the humans (Shi et al., 2015). One of the major goals of goat farming is to improve milk quality through the alteration of milk composition, according to specific needs of target groups such as infants or immune-compromised individuals (Wickramasinghe et al., 2012). To achieve this goal, a thorough understanding of milk components and their regulatory factors is required.

The mammary gland has been widely-used as a research model to investigate the mechanisms of milk formation and secretion (Shennan and Peaker, 2000). However, it was not until the mid-2000s that the first study utilizing large-scale transcriptome profiling was published (Rudolph et al., 2003). That original study, although broad in its focus, and a subsequent one underscored that biosynthesis and secretion of lipid in mammary gland tissue is regulated by complex gene networks (Rudolph et al., 2007; Andres and Djonov, 2010). Subsequent work in dairy cows focusing on a curated list of 45 genes associated with lipid synthesis (triacylglycerol and phospholipids) and secretion reported the first longitudinal profiles from late pre-partum to the end of subsequent lactation (Bionaz and Loor, 2008). These observations allowed for the development of a network of genes participating in the coordination of milk fat synthesis and secretion.

Despite the substantial body of work in bovine, research on the mammary gland of small ruminant species, particularly in goats, is limited. For instance, most published studies on goats have focused on functional verification of candidate genes associated with lipid metabolism using data from human, mouse and cattle, including *SCD1* (Yao et al., 2017), *SREBP1* (Xu et al., 2016), *FASN* (Zhu et al., 2014), *INSIG* (Li et al., 2019), and *ELOVL7* (Shi et al., 2019). Although those efforts have enhanced our knowledgebase, economically-important traits in livestock such as milk lipid synthesis and composition are genetically affected by some causal genes together with many genes with minor effects, which together result in complex regulatory networks (Goddard and Hayes, 2009; Georges et al., 2019). Extensive and detailed molecular research in dairy cows has underscored that the ruminant mammary gland is a very dynamic organ with remarkable plasticity (Akers, 2017), especially during the transition from pregnancy to lactation. However, there is a paucity of data regarding changes occurring in the goat mammary gland during the physiological transition from established lactation to dry-off to involution. Therefore, for a better understanding of regulatory mechanisms underlying milk fatty acid metabolism and lactation in dairy goats, a systematic screen of gene expression profiles is needed.

Development of high-throughput sequencing has made it feasible to generate a whole genome sequence in the domestic goat (Mardis, 2008; Metzker, 2010). In addition, mining the differentially expressed genes (DEGs) in mammary tissue across discrete stages of lactation could vastly expand our knowledge of the molecular regulation of mammary gland function in small ruminants. Therefore, in the present study we used Illumina digital gene expression (DGE) profiling sequencing to characterize transcript profiles in mammary gland tissue across 3 stages of the lactation cycle in Xinong Saanen dairy goats. Beyond expanding our mechanistic knowledge, identifying novel promising genes responsible for the genetic variation could contribute greatly to our understanding of lactation and milk fatty acid metabolism. It could help in the future to develop a molecular breeding program with the aim of improving fatty acid composition in dairy goat milk.

## Materials and Methods

### Ethics Statement

The present experiment was conducted in accordance with approved guidelines by the Animal Care and Use Committee of the Northwest A&F University. The committee approved all procedures and experiments with live animals.

### Goat Mammary Gland Collection and RNA Extraction

Mammary gland tissue was collected based on methods described previously (Shi et al., 2015). Briefly, a total of nine healthy Xinong Saanen dairy goats (approximately 3 to 4 years old from 2 to 3 parities) were selected from the experimental farm at Northwest A&F University (Shaanxi, China). All goats were managed similarly and were fed a mixed diet comprised of corn, soybean meal, bran, rapeseed meal and mineral-vitamin premix. Approximately 2 g mammary gland tissue was collected from the mid-region of the right mammary gland of each goat at peak lactation (3 goats, 100 days postpartum; L group), dry-off (cessation of milking, 3 goats, 310 days postpartum; D group) and non-lactating/non-pregnant period (involution, 3 goats; NP group). All tissue samples were obtained under sterile conditions on the same day, harvested within 20 min for each and immediately frozen in liquid nitrogen until RNA extraction.

Total RNA extraction, mRNA purification, and cDNA library construction were conducted by LC Sciences (Houston, TX, United States). In brief, total RNA was obtained from mammary gland tissue using a total RNA purification kit (LC Sciences, Houston, TX, United States), treated with RNAase-free DNAase, and re-purified with the RNA easy kit (Qiagen, Valencia, CA, United States) following the manufacturer’s instructions. Total RNA quantity and quality were analyzed with the RNA 6000 Nano LabChip Kit in the Bioanalyzer 2100 system (Agilent Technologies, CA, United States).

### cDNA Library Construction and Illumina Sequencing

The mRNA was purified from total RNA using poly-T-oligo-attached magnetic beads (Invitrogen, United States). Following purification, the mRNA was fragmented into small pieces using divalent cations at a high temperature. After phosphatase and polynucleotide kinase (PNK) treatment, the cleaved mRNA fragments were reverse-transcribed. The 200–300 bp fragments were purified using 6% polyacrylamide Tris-borate-EDTA to create the final cDNA library. One end sequencing of 1 × 36 bp was then performed on an Illumina HiSeq 2000 platform following the vendor’s recommended protocols. The application of DGE sequencing to uncover DEGs has advantages such as low cost, rapid analysis, and high accuracy (t Hoen et al., 2008).

### Data Filtering and Expression Level Determination

The raw reads were first determined by fastQC^1^ and filtered by removing potential contamination, reads with unknown base more than 1 N (base not available), and low quality sequences (parameter -q 36 -p 90) using the Fastx_toolkit (0.0.13) (Lohse et al., 2012). All short reads were assembled as transcripts using Trinity software (Haas et al., 2013). As a subset of the transcript, unigene was the longest transcript of each cluster that matched the same reference gene. HTSeq v0.6.1 was used to count the reads numbers mapped to each unigene. By aligning sequences with a goat database constructed previously (BioProject ID: PRJNA243005), the gene expression level was recorded according to the sum frequencies of compared reads. Reads per kilo base of exon model per million mapped reads (RPKM) were used for expression normalization, and the gene expression RPKM values were categorized into three groups: high (≥500 RPKM), medium-to-high (10 to 500 RPKM) and low (≤10 RPKM) (Wickramasinghe et al., 2012; Canovas et al., 2014). Assembled unigenes were annotated to the reference dataset using BLAST. Databases including a manually Annotated and Reviewed Protein Sequence Database (SWISS-PROT), NCBI non-redundant protein sequence database (NR), Pfam database, KOG, GO, and KEGG were performed with an E-value cutoff of 1E-10 according to previously published studies (Ogata et al., 1999; Shi et al., 2015).

### Analysis of DEG Data

The normalized ratio of the gene expression signals was log_2_ transformed. The RVM (Random variance model) N^2^-test and Chi-square test were applied to filter DEGs. After analyses for statistical significance and FDR (false discovery rate), DEGs were selected according to *P* < 0.05 and FDR < 0.05 (Wright and Simon, 2003; Yang et al., 2005; Clarke et al., 2008). Bowtie 0.12.8 was used for abundance analysis of gene expression. Short Time-series Expression Miner (STEM) was used for expression pattern analysis (Ernst and Bar-Joseph, 2006). Using the log normalized data method, the “maximum number of model profiles” was set at 20, and the “maximum unit change in model profiles between time points” was set at 2 (Ernst and Bar-Joseph, 2006). Gene co-expression analysis (Pujana et al., 2007; Xu et al., 2013) was performed to track interactions among DEGs. Pearson correlation analysis was performed for each pair of genes, and significantly correlated pairs were used to construct the network with a threshold of 0.92 using a Perl script (Prieto et al., 2008). The number of correlated genes was used for node gene selection. Based on all DEGs identified when adding up the three pairs of comparisons (L vs. D, L vs. NP, D vs. NP), potential candidate genes regulating lactation and milk fatty acid metabolism were identified.

### Dynamic Impact Approach (DIA) Analysis

Data were filtered with the threshold at fold-change >2 and *P* < 0.001, and were mined through an integrative systems biology approach applying the DIA method (Shi et al., 2017). The bovine KEGG pathways and GO biological process category database were used for functional analysis with the DIA. Detailed methodology for data analysis using DIA was described previously (Bionaz et al., 2012b). The estimate of the perturbation in a biological pathway is represented by the “Impact” while the overall direction of the perturbation is represented by the “Flux” (or direction of the impact) (Shi et al., 2017). The impact was obtained by combining the proportion of DEGs with the log_2_ mean fold-change and mean –log *P*-value of genes associated with the biological term. The direction of the impact was calculated as the difference of the impact of up-regulated DEGs and downregulated DEGs associated with the biological term (Bionaz et al., 2012b). The analytical flow chart used in the present study is described in Supplementary Figure S1.

## Results

### Illumina Sequencing and Gene Annotation

**FIGURE 1:**
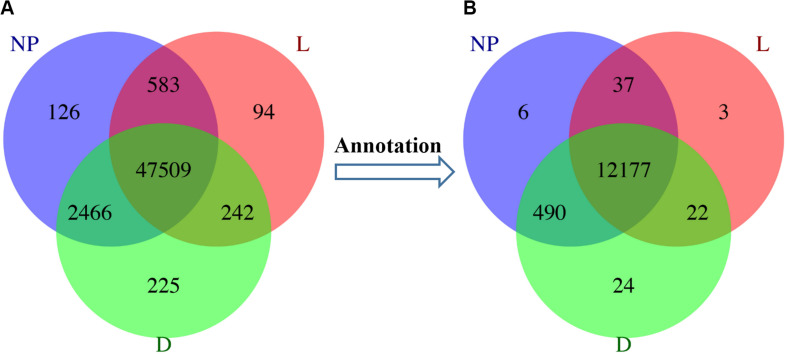
Unigene distribution in goat mammary gland during different stages of lactation. **(A)** Unigenes distributed in peak lactation (L), dry-off (D) and non-lactating/non-pregnant (NP) periods. **(B)** The distribution of functional genes during different stages of lactation.

A total of 63,256,236 clean reads were obtained with an average of 7,028,470 (range from 3,423,245 to 10,365,063 reads) for each sample. After sequence assembly according to the goat transcriptome sequence published in our previous study (Shi et al., 2015), out of 98,864 transcripts (Supplementary File S2A) the Illumina sequencing uncovered a total of 51,299 unigenes. Among these unigenes, 48,428, 50,442, and 50,684 unigenes were associated with the lactation groups “L,” “D,” and “NP” respectively. The number of unigenes uniquely expressed in the “L,” “D,” and “NP” groups were 94, 225, and 126, respectively (Figure 1A). Importantly, a total of 12,763 genes were identified through annotation, with 12,239, 12,713, and 12,710 genes in the “L,” “D,” and “NP” group, respectively. Among those identified genes, 3, 24, and 6 genes, respectively, were uniquely expressed in each group (Figure 1B).

### Lipid Biosynthesis and Metabolism-Associated Genes

Gene expression level was evaluated using the RPKM method. Goats in the “L” group expressed a total of 12,239 genes including 65 with the RPKM greater than 500, 1,102 genes with RPKM between 10 and 500, but most genes (11,072) had expression below 10 RPKM. According to the same RPKM classification, out of a total of 12,713 and 10,710 genes in the “D” and “NP” group there were 104 and 77 genes with RPKM greater than 500, 3,470 and 2,262 genes with RPKM between 10 and 500, while 9,139 and 10,371 genes had expression below 10 RPKM.

The most abundant genes were further filtered by focusing on fatty acid biosynthesis and metabolism as well as lipid transport and metabolism pathways or terms using the KOG, GO, and KEGG categories. Of these, the 15 most abundant genes related to lipid transport and metabolism are reported in Supplementary Table S1. *FASN*, *FABP4*, *ACBP*, *EBP*, *RNPEPL1*, *DEGS1*, and *SAPOSIN* were detected in all three stages of lactation. The most abundant genes involved in intracellular trafficking, secretion, and vesicular transport were *SSR4*, *lepB*, *SEC61G, AP2M1*, *ARF1*, *CHMP2A*, *CLTA*, *RAB11B*, *RAB1A*, *SDHD*, and *SEC61* (Supplementary Table S2).

Within the GO fatty acid metabolism category, *EHHADH* was the most abundant gene in lactation stages “L,” “D,” and “NP,” with RPKM of 24.134, 34.567, and 34.016, respectively. Well-known genes involved in fatty acid metabolism such as *ACAA2*, *ACSL* and *ACAA* had a medium-to-high expression level across all lactation stages (Supplementary Table S3). Within fatty acid biosynthetic process in the GO category, *FASN* was the most abundant gene across lactation stages with 376.222, 120.146, and 537.391 RPKM in “L,” “D,” and “NP,” respectively. In addition, the most abundant genes observed in all three lactation stages were *SCD*, *ELOVL1*, *DEGS1*, *NADH*, *NDUFAB1*, *ABDH2*, *MSMO1*, *PRKAG1*, *SC5D*, *STK11*, and *TECR* (Supplementary Table S4).

For genes involved in fatty acid metabolism signaling pathways revealed by KEGG, *ACAA2*, *ACSL*, *ACADL*, *ACADM*, *ACADS*, *ACADSB*, *ACOX1*, *ACAT1*, *ADH1*, *ALDH1*, *CPT1A*, *CPT1B*, and *GCDH* were the most abundant genes detected across lactation stages (Supplementary Table S5). Within fatty acid biosynthesis signaling pathway identified through KEGG, *FASN*, *HLCS*, *MCAT*, and *OXSM* were the top four most abundant genes across lactation stages (Supplementary Table S6).

### Identification of Differential Gene Expression Patterns

**FIGURE 2:**
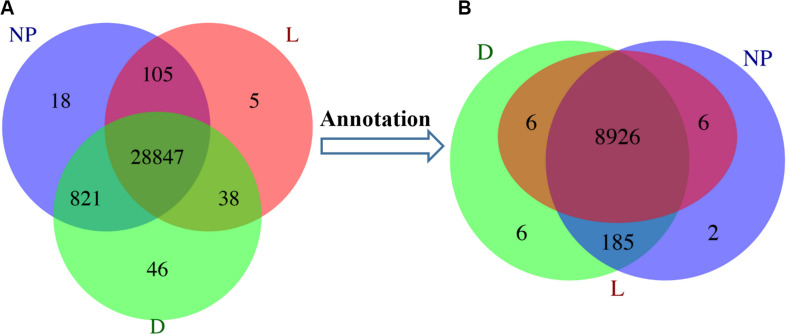
Distribution of DEGs during different stages of lactation. L, peak lactation period; D, dry-off period; NP, non-lactating/non-pregnant period. **(A)** Differentially expressed unigenes distributed in peak lactation (L), dry-off (D) and non-lactating/non-pregnant (NP) periods. **(B)** Annotation of DEGs during different stages of lactation.

A total of 29,880 differentially expressed unigenes were detected among different groups, annotated as 9,131 DEGs (Supplementary File S2B). In the “D” and “NP” groups, a total of 46 and 18 unique unigenes were annotated to 6 and 2 DEGs, respectively. Although five uniquely expressed unigenes were detected in the “L” group, they were not annotated to any functional gene (Figure 2). A total of eight unique DEGs across lactation stages are reported in Table 1.

**TABLE 1:** Uniquely expressed DEGs in each lactation stage in dairy goat mammary gland tissue.

Number	Accession	Gene symbol	Description	RPKM	Stage
1	comp30822_c1_seq1	BMP-3B	bone morphogenetic protein 3/3B	0.415	NP
2	comp26554_c0_seq1	GPR64	G protein-coupled receptor 64	0.386	NP
3	comp1343_c1_seq1	MYSM1	protein MYSM1	0.958	D
4	comp14291_c0_seq1	MYB	myb proto-oncogene protein	1.720	D
5	comp15472_c0_seq1	GZMH	granzyme H (cathepsin G-like 2)	5.420	D
6	comp17324_c0_seq1	GJB2	gap junction protein, beta 2	2.511	D
7	comp25676_c0_seq1	ITGA11	integrin alpha 11	0.659	D
8	comp28858_c0_seq1	ZSCAN20	KRAB domain-containing zinc finger protein	0.821	D

*“NP” represents non-lactating/non-pregnant period; “D” represents dry-off period.*

**FIGURE 3:**
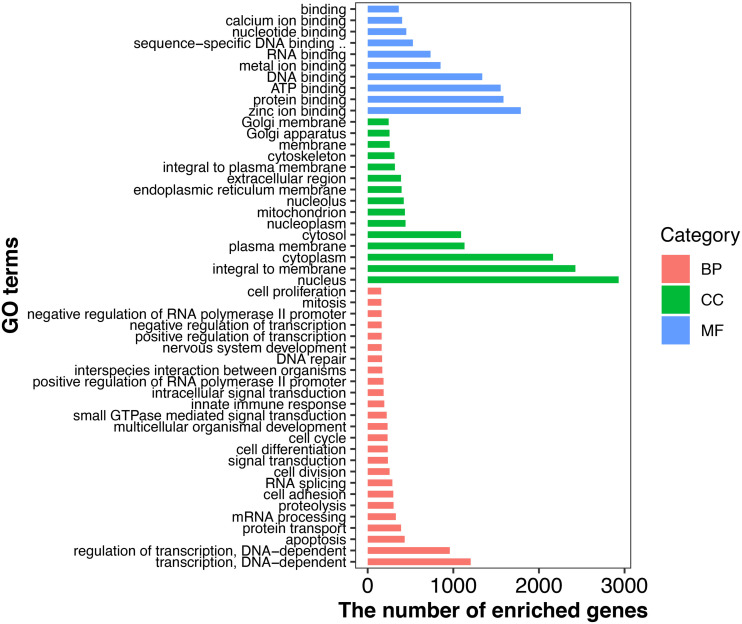
GO classifications of DEGs in goat mammary gland. Distribution of the GO categories assigned to the goat mammary gland transcriptome. DEGs were classified into three categories: biological processes, cellular components, and molecular functions. L, peak lactation period; D, dry-off period; NP, non-lactating/non-pregnant period.

In total, 50 GO terms including biological process, cellular component and molecular function were significantly enriched with 8,311 DEGs (Figure 3). Of these, transcription, DNA-dependent and regulation of transcription had the most-abundant DEGs across 25 enriched biological processes. With 15 terms in the cellular component category, most DEGs were related to the nucleus, integral to membrane, and cytoplasm. A total of 10 terms involved in molecular function including zinc ion binding, ATP binding, and protein binding had the most DEGs (Figure 3). In addition, a total of 246 KEGG pathways were enriched 5,048 DEGs (Supplementary File S2C) with Insulin signaling, Jak-STAT signaling, Glycerophospholipid metabolism, mTOR signaling and PPAR signaling among the most-relevant to lactation and milk component synthesis regulation.

The most abundant DEGs involved in fatty acid biosynthesis and metabolism processes, lipid metabolism, protein metabolism, and lactose metabolism were used to mine within GO and KEGG categories for pathways or terms across lactation stages (Supplementary Tables S7–S10). A total of 48 DEGs were identified in terms of fatty acid metabolic processes/pathways, whereas 18 DEGs including *ARL5A*, *MARK*, *STAM*, and *EPHA1* were simultaneously revealed by GO and KEGG (Supplementary Table S7). Among a total of 36 identified DEGs associated with fatty acid biosynthetic processes/pathways, *SEMA6*, *RUNX1T1*, and *LEF1* were detected by both GO and KEGG (Supplementary Table S7). Within GO and KEGG, the well-known genes *GPAT3*, *ADH* and *ALDH* involved in lipid metabolic processes/pathways were among a total of 29 most abundant DEGs (Supplementary Table S8). Highly expressed DEGs such as *NR1F1*, *EIF3S5*, *MYH*, and *ABCF2* were in the lipid biosynthetic processes/pathways (Supplementary Table S8).

For protein metabolism, 4 DEGs were identified in protein biosynthetic processes, 21 DEGs were grouped in protein metabolic process, including *STAT5A*, *IGF1*, *IGFR2*, and *MARCH2*. Additionally, 15 DEGs were identified within protein export pathway, including *GPAT3*, *NR1F1*, and *IGFR2* (Supplementary Table S9). *B4GALT5*, *SERPINE1*, and *MDH2* were identified as the top-most abundant DEGs in the lactose metabolic process/pathway (Supplementary Table S10).

**FIGURE 4:**
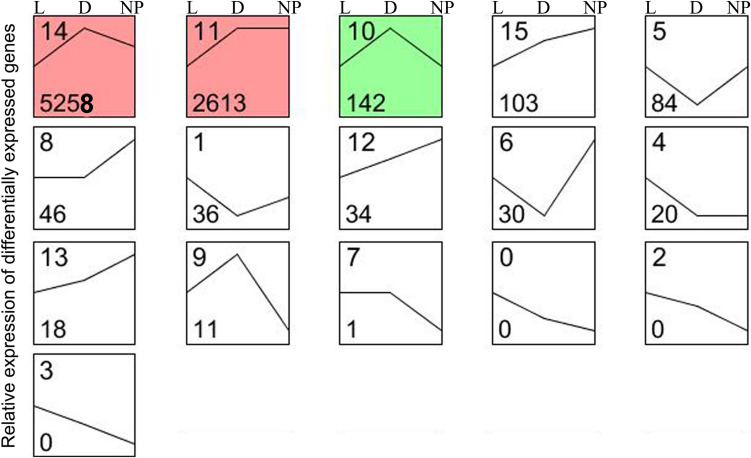
Sixteen patterns encompassing the DEGs during different stages of lactation using STEM software. L, peak lactation period; D, dry-off period; NP, non-lactating/non-pregnant period. Clusters ordered based on number of genes, and profiles ordered by significance. The number in the top left-hand corner of a profile box is the profile ID number, and the number in the bottom left-hand corner of a profile box is the profile gene number. The colored profiles had a statistically significant number of genes assigned. Non-white profiles of the same color represent profiles grouped into a single cluster.

A total of 16 possible expression patterns were detected among the 9,131 DEGs (Figure 4 and Supplementary File S2D). Of these, seven patterns (9, 10, 11, 12, 13, 14, and 15) containing 8,179 DEGs were upregulated in stage of lactation “D” compared with “L” (*P* < 0.05). In contrast, seven patterns (0, 1, 2, 3, 4, 5, and 6) representing 170 DEGs were downregulated in lactation stage “D” (*P* < 0.05). The other two patterns (7 and 8) representing 47 DEGs had a similar expression level in the “D” and “L” groups. A total of seven patterns (1, 5, 6, 8, 12, 13, and 15) representing 351 DEGs were upregulated in lactation stage “NP” compared with “D” (*P* < 0.05), whereas, another seven patterns (0, 2, 3, 7, 9, 10, and 14) representing 5,409 DEGs were significantly downregulated in the “NP” group compared with “D” group. The patterns 11 and 4 containing 2,633 DEGs had the similar expression levels between the “D” and “NP” group.

Co-expression analysis revealed that 13 DEGs interacted with 8,028 correlated background genes including *POLG*, *SPTA1*, *KLC*, *GIT2*, *COPS3*, *PDP*, *CD31*, *USP 16/29/37*, *TLL1*, *NCAPH*, *ABI2*, *DNAJC4*, and *MAPK8IP3* (Table 2). Specifically, three genes involved in lipid transport and metabolism (*PLA2*, *CPT1*, and *PLD*) had the highest number of correlated genes (Table 3). In addition, threegenes had associations with intracellular trafficking, secretion, and vesicular transport with *GGA*, *SRPRB*, *AP4S1* having the greatest number of correlated genes (Table 4). The correlated genes for node gene selection are reported in Supplementary File S2E.

**TABLE 2:** Detailed information of 13 novel candidate genes that are putative regulators of lactation.

Number	Accession	Number of correlated genes	Description	Gene name	Expression pattern serial number	*P* value
1	comp46002_c1_seq12	8028	DNA polymerase gamma 1	POLG	14	0.0001
2	comp45782_c0_seq12	8028	spectrin alpha	SPTA1	14	0.0003
3	comp45470_c0_seq9	8028	kinesin light chain	KLC	14	0.0056
4	comp44279_c0_seq18	8028	G protein-coupled receptor kinase interactor 2	GIT2	14	0.0105
5	comp42963_c1_seq1	8028	COP9 signalosome complex subunit 3	COPS3	14	0.0006
6	comp38172_c0_seq1	8028	pyruvate dehydrogenase phosphatase	PDP	14	0.0105
7	comp35137_c0_seq1	8028	platelet/endothelial cell adhesion molecule	CD31	14	0.0152
8	comp347610_c0_seq1	8028	ubiquitin carboxyl-terminal hydrolase 26/29/37	USP16/29/37	11	0.0087
9	comp30737_c0_seq1	8028	tolloid-like protein 1	TLL1	14	0.0267
10	comp27926_c0_seq1	8028	condensin complex subunit 2	NCAPH	14	0.0151
11	comp260385_c0_seq1	8028	abl interactor 2	ABI2	14	0.0125
12	comp21168_c0_seq1	8028	DnaJ homolog, subfamily C, member 4	DNAJC4	14	0.0069
13	comp18849_c0_seq1	8028	mitogen-activated protein kinase 8 interacting protein 3	MAPK8IP3	14	0.0449

**TABLE 3:** Significant node DEGs involved in lipid transport and metabolism in the KOG database.

Number	Accession	Number of correlated genes	Description	Gene name	Expression pattern serial number	*P* value
1	comp14302_c0_seq1	8027	phospholipase A2	PLA2	14	0.0000
2	comp37635_c0_seq1	8025	choline-phosphate cytidylyltransferase	CPT1	14	0.0032
3	comp3416_c1_seq1	8022	phospholipase D	PLD	14	0.0027
4	comp81055_c0_seq1	8018	phospholipase C, beta	PLCB2	14	0.0029
5	comp44474_c0_seq3	8018	acyl-CoA thioesterase 8	ACOT8	14	0.0016
6	comp44308_c1_seq5	8018	phosphatidate phosphatase	LPIN1	14	0.0156
7	comp41695_c0_seq1	8018	myo-inositol-1-phosphate synthase	MIPS	14	0.0031
8	comp19736_c0_seq1	8018	lysophospholipase	PLB1	11	0.0002
9	comp109409_c0_seq1	8018	ATP-binding cassette, subfamily D (ALD), member 1	ABCD1	14	0.0041
10	comp106085_c0_seq1	8018	cytochrome P450, family 4, subfamily B	CYP4B1	14	0.0001
11	comp42853_c0_seq50	8013	acid phosphatase	ACP	14	0.0027
12	comp45100_c0_seq4	8011	phospholipase C, gamma	PLCG1	14	0.0002
13	comp41725_c0_seq1	8010	phosphatidylserine synthase 2	PTDSS2	14	0.0272
14	comp35037_c0_seq1	8009	estradiol 17beta-dehydrogenase	HSD17B1	14	0.0005
15	comp30693_c0_seq1	8008	sterol O-acyltransferase	SOAT1	14	0.0116

**TABLE 4:** Significant node DEGs involved in intracellular trafficking, secretion, and vesicular transport in the KOG database.

Number	Accession	Number of correlated genes	Description	Gene name	Expression pattern serial number	*P* value
1	comp40427_c0_seq1	8027	ADP-ribosylation factor-binding protein GGA	GGA	14	0.0075
2	comp42365_c0_seq1	8025	signal recognition particle receptor subunit beta	SRPRB	14	0.0005
3	comp270281_c0_seq1	8023	AP-4 complex subunit sigma-1	AP4S1	11	0.0030
4	comp30233_c1_seq1	8022	DnaJ homolog, subfamily C, member 13	DNAJC13	14	0.0001
5	comp38721_c0_seq1	8021	charged multivesicular body protein 3	CHMP3	14	0.0049
6	comp18653_c0_seq1	8021	serum/glucocorticoid regulated kinase	SGK1	14	0.0013
7	comp42059_c0_seq14	8020	exocyst complex component 3	EXOC3	14	0.0002
8	comp69910_c0_seq1	8019	AP-2 complex subunit alpha	AP2A1	14	0.0454
9	comp44186_c0_seq6	8019	dynamin GTPase	DNM1	14	0.0005
10	comp91177_c0_seq1	8017	ATP-binding cassette, subfamily B (MDR/TAP), member 7	ABCB7	14	0.0029
11	comp42215_c0_seq4	8017	vesicle-associated membrane protein 5	VAMP5	14	0.0016
12	comp40781_c0_seq1	8017	Ras-related protein Rab-5A	RAB5A	14	0.0007
13	comp20400_c0_seq1	8017	vesicle-associated membrane protein 7	VAMP7	14	0.0010
14	comp44474_c0_seq4	8016	acyl-CoA thioesterase 8	ACOT8	14	0.0011
15	comp33721_c0_seq2	8012	Ras-related protein Rab-4B	RAB4B	14	0.0105

### DIA Analysis of DEGs

**FIGURE 5:**
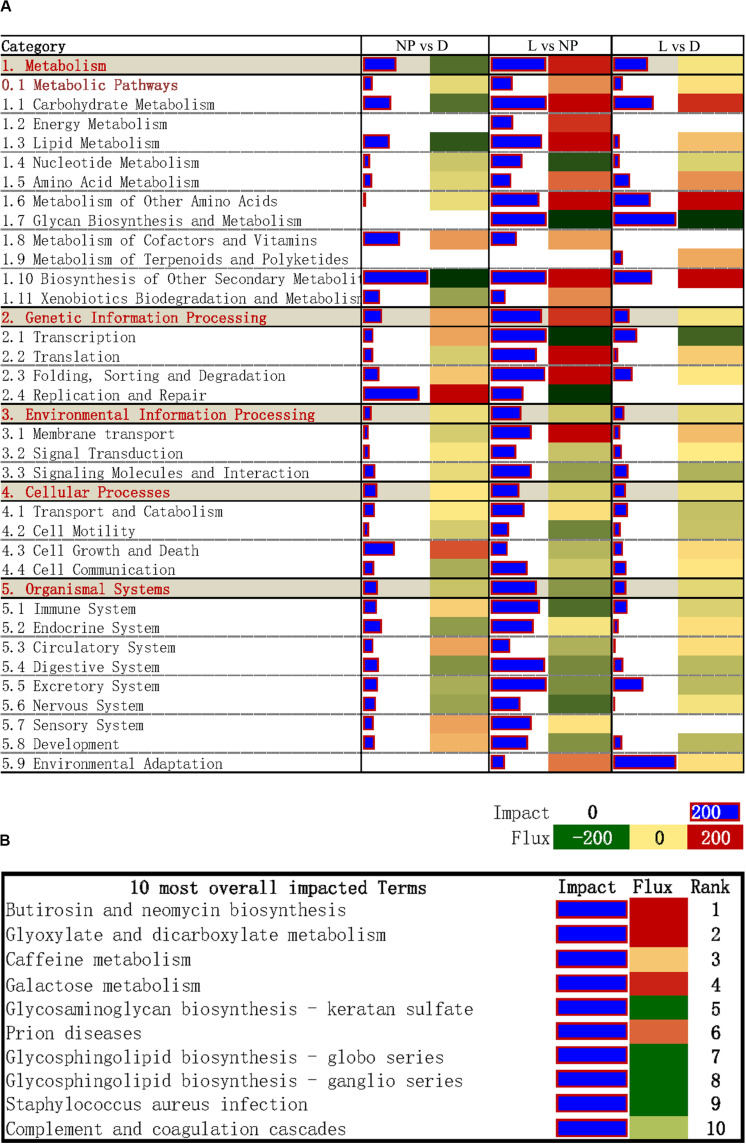
DIA analysis of DEGs during different stages of the lactation cycle. **(A)** The summary of KEGG pathways provided by DIA. The “impact” is represented by the horizontal blue bars (the larger the bar, the larger the impact) and the “flux” (Direction of the Impact) is represented by green (more inhibited) to red (more activated) rectangles. **(B)** DIA results for the 10 most impacted KEGG pathways.

The DIA provides a summary of the KEGG pathways in the form of categories and sub-categories (Figure 5A) that are altered across a given comparison. Details of each pathway are presented in Supplementary File S3. As shown in Figure 5A, KEGG pathway categories were more impacted in stage of lactation “L” versus “NP.” Among these pathways, the category “Metabolism” was the most impacted one, followed by the category “Genetic Information Processing.” With the exception of the subcategory of pathways within “Metabolism of Terpenoids and Polyketides,” all other subcategories within Metabolism had an impact value >50 in the comparison of “L” versus “NP,” with highest impact value (880) in “Biosynthesis of Other Secondary Metabolites.” Except for inhibition of “Glycan Biosynthesis and Metabolism” and “Nucleotide Metabolism,” compared with “NP,” most of the metabolic pathways were markedly activated in the “L” group including “Carbohydrate Metabolism,” “Energy Metabolism,” “Lipid Metabolism,” “Amino Acid Metabolism,” “Metabolism of other Amino Acid,” “Metabolism of Cofactors and Vitamins,” and “Xenobiotics Biodegradation and Metabolism.” According to the impact value, the categories “Environment Information Processing,” “Cellular Process,” “Organismal System” were also altered in the comparison of “L” versus “NP.” However, most of their flux values were slightly activated or did not change in the comparisons of “D” versus “NP” or “L” versus “NP.”

The top 10 overall most-impacted terms are shown in Figure 5B. The most impacted pathway was “Butirosin and neomycin biosynthesis” followed by “Glyoxylate and dicarboxylate metabolism” with flux >200 (Figure 5B). The categories “Galactose metabolism” were highly activated. In contrast, the pathways “Glycosaminoglycan biosynthesis-keratan sulfate,” “Glycosphingolipid biosynthesis - globo series,” and “Glycosphingolipid biosynthesis-ganglio series” were inhibited.

## Discussion

The development of next-generation sequencing has made it feasible to explore patterns of DEGs in goat mammary glands across stages of lactation. Similar to bovine, this information will help us better understand fatty acid metabolisms and tissue function at the transcriptional level (Bionaz et al., 2012a). One limitation of the current experiment was that mammary gland tissue harvested at the three stages of lactation was not from the same goats, which might overshadow the specific effects associated with lactation. A similar experimental approach for tissue collection and analyses has been utilized in previous work with dairy cows (Li et al., 2016; Cai et al., 2018). However, unlike dairy cows with a large udder mass, repeated mammary biopsies in small ruminants is riskier and prone to inducing cessation of lactogenesis. The fact that peak lactation in the goat occurs at ∼100 days postpartum meant that biopsies collected at dry-off would have taken place roughly 200 days afterward, which was deemed problematic due to the uncertainty about recovery from a biopsy. From a technical standpoint, sampling different animals at the target stages of lactation within the same day increased accuracy of sequencing partly because of the characteristics of temporality and spatiality of mRNA expression.

In the present study, we identified DEGs across three important stages of the lactation period, i.e., established lactation, dry-off, and the non-lactating/non-pregnant period. Data mining revealed key factors involved in the control of mammary gland function, as well as improved our understanding about the role of transcriptional regulation in coordinating lactation. The assembly of 98,864 transcripts was greater than that in macaque (42,702 transcripts) (Gao et al., 2013), bovine (35,945 transcripts) (Shen et al., 2015), and pig (21,331 transcripts) (Zhu et al., 2014). The fact that the latest reference genome was not used in the present study may be a potential reason for the greater number of transcripts detected. However, an advantage of the approach we used is that we could perform a better mapping of the sequences with a more robust unigene annotation. The sequences in this study were assembled according to the goat transcriptome sequences published in our previous study (Shi et al., 2015), i.e., the first study to characterize the complete transcriptome of goat mammary gland, constituting a comprehensive genomic resource available for studies of ruminant lactation. An N50 length is commonly used for assembly evaluation, and a higher number suggests higher-quality assembly (Lander et al., 2001). The N50 length of assembly in our previously published transcriptome sequences (Shi et al., 2015) was higher than other ruminant transcriptome data with a higher overall number of unique transcripts (Wickramasinghe et al., 2011, 2012), suggesting it could provide a comprehensive reference dataset of gene expression profiling for future goat mammary gland research.

It is well known that *FASN* is a crucial gene for *de novo* fatty acid synthesis in mammary cells (Zhu et al., 2014). Consistent with our previous qRT-PCR analysis, the expression of *FASN* in mammary tissue of goats was higher during lactation than dry-off (Zhu et al., 2014). This may partly explain the enrichment of short- and medium-chain fatty acids in goat milk (Morris et al., 2007; Silanikove et al., 2010). However, the up-regulation of *FASN* in the non-lactating/non-pregnant period, despite the shutting down of lactogenesis within mammary cells, was somewhat unexpected. It is possible that this response was related to an additional need of fatty acids for cell proliferation (Mellenberger et al., 1973) associated with mammary gland development during pregnancy (Faucon et al., 2009), which would be important for the turnover of mammary gland (Norgaard et al., 2008).

Although determining differences between lactating and non-lactating periods has helped elucidate regulatory mechanisms that are controlling lactation (Bionaz and Loor, 2008; Li et al., 2012; Wickramasinghe et al., 2012), it is also important to uncover the DEGs that are specifically induced during pregnancy (Faucon et al., 2009). In the present study, in order to avoid the effects of pregnancy (e.g., endocrine status) on gene expression, we examined expression trends of DEGs among the three periods and detected 20 DEGs with lower and stable expression during the non-lactating and non-pregnant period compared with lactation (Figure 4, pattern 4). Although some milk fat-related genes might be important during pregnancy (Linzell, 1959), most of the genes related to milk fat formation had expression levels that were between those observed in the dry-off (pregnant) and the non-lactating (non-pregnant) periods. More functional research is needed for identifying the role of these genes.

A total of two and six DEGs were determined as uniquely expressed in the dry-off and non-lactating periods. In the non-lactating group, bone morphogenetic protein 3/3B (*BMP-3B*) is a cytokine that belongs to the transforming growth factor β (*TGF-*β) superfamily, and plays an important role in the antral nervous system as well as in bone formation and remodeling (Takao et al., 1996). The G protein-coupled receptor 64 (*GPR64*) is well-known as a member of G protein-coupled receptor superfamily, and is crucial for neuronal development regulation (Richter et al., 2013). Despite putative functions in development, their expression in the non-lactating period was relatively low (RPKM were 0.415 and 0.386, respectively). These results are in close agreement with the extensive remodeling of the mammary gland during the non-lactating period (McDaniel et al., 2006; Safayi et al., 2010).

Among genes with higher expression in the dry-off group, protein Myb-Like, SWIRM and MPN domains 1 (*MYSM1*) are important for the control of B cell development (Jiang et al., 2011). Myb proto-oncogene protein (*MYB*) promotes proliferation and inhibits differentiation (Hugo et al., 2013) and also has a key role in regulating stem and progenitor cells in the bone marrow, colonic crypts and a neurogenic region of the adult brain (Ramsay and Gonda, 2008). The gap junction protein beta 2 (*GJB2*) is important for the development of the auditory system (Bozdogan et al., 2015). All these genes had a relatively higher expression level (the RPKM was 0.958, 1.720, and 2.511, respectively) and most of them are beneficial for embryo development, probably playing a role in mammary cells during dry-off to prepare well in preparation for the impending parturition.

Co-expression analysis is a suitable method for regulatory gene selection (Xu et al., 2013). A node gene correlated with the greatest numbers of genes was considered crucial for the regulation of lactation. Our analysis revealed that *POLG*, *SPTA1*, *KLC*, *GIT2*, *COPS3*, *PDP*, *CD31*, *USP 16/29/37*, *TLL1*, *NCAPH*, *ABI2*, *DNAJC4*, and *MAPK8IP3* correlated with most genes. In turn, these genes may play roles in regulating mammary cell function in the lactation stages that were examined in the current study.

Replication of DNA and repair in mitochondria is a crucial function of *POLG*. *SPTA1* is the major protein component of the erythrocyte membrane skeleton (Huebner et al., 1985). *KLC* is a component of Kinesin heterotetramer and acts in binding motor protein to cargo (Hanks et al., 1988). *GIT2* plays an important role in cell attachment, spreading and motility (Pahlich et al., 2006). *COPS3* is essential for maintenance of cell proliferation in the mouse embryonic epiblast (Buhr et al., 2008). *PDP* is important for dephosphorylating pyruvate dehydrogenase in the mammalian pyruvate dehydrogenase complex which catalyzes the conversion of pyruvate to acetyl-CoA, a substrate for *de novo* fatty acid synthesis (Gonsalvez et al., 2007). *CD31* is associated with the vascular compartment and is uniquely-positioned to mediate multiple and important cell-cell interactions involving platelets, leukocytes and endothelial cells (Zheng et al., 2005). *USP* is essential for maintaining the structure and function of neuromuscular junction (Verbiest et al., 2008). *TLL1* is necessary for normal septation and positioning of the heart (Thomassen et al., 2009). *NCAPH* plays an important in regulating cell cycle activity and proliferation (Hardwick, 2008). *ABI2* is involved in abscisic acid signal transduction in Arabidopsis (Dean et al., 2001). *DNAJC4* is a sterol-regulatory element binding protein (*SREBP*)-regulated chaperone involved in the cholesterol biosynthesis pathway. *MAPK8IP3* is involved in c-Jun N-terminal kinase (JNK) signaling pathway.

All the above genes have a wide number of functions associated with cellular regulation, consistent with the idea that the process of lactation is regulated and balanced by a complex network of genes (Bionaz and Loor, 2008). Consistent with previous studies, the node genes are central for lipid transport and metabolism along with intracellular trafficking, secretion, and vesicular transport. Specifically, *PLA2* (Zhu et al., 2011; Shen et al., 2012; Zhang et al., 2013), *CPT1* (Bovia et al., 1995), *GGA* (Gustavsson et al., 2014), and *SRPRB* (Hill et al., 1988) had the most number of correlated genes. Although the exact role of these genes in fatty acid metabolism during the various stages of lactation remains to be determined, they may represent candidate genes predicted to be beneficial for our greater understanding of milk fat synthesis.

By combining the proportion of DEGs with the log_2_ mean fold change and mean -log *P*-value of genes associated with the biological terms, the DIA is a useful approach to estimate the biological impacts of experimental conditions and the direction of impact (Shi et al., 2017). It has been successfully used for studying goat mammary lipid metabolism in isolated epithelial cells (Lin et al., 2013). In the present study, we determined “Glyoxylate and dicarboxylate metabolism” as one of the most-impacted metabolic pathways during lactation, and was closely associated with the biosynthesis of acetyl-CoA, the substrate for *de novo* fatty acid synthesis. Thus, the present results underscored alterations of milk lipid synthesis during lactation in goat mammary gland as one of the most-important functional aspects occurring in this organ.

## Conclusion

The comprehensive transcriptome profiling of mammary gland across different stages of lactation in Xinong Saanen dairy goat revealed a total of 51,299 unigenes that were annotated to 12,763 genes. The most highly expressed genes were involved in milk fatty acid synthesis and metabolism. Eight out of 9,131 identified DEGs were uniquely expressed in the non-lactating or dry-off periods. We obtained 16 possible expression patterns among DEGs and detected 13 node genes potentially regulating functional adaptations across stages of lactation: *POLG*, *SPTA1*, *KLC*, *GIT2*, *COPS3*, *PDP*, *CD31*, *USP16/29/37*, *TLL1*, *NCAPH*, *ABI2*, *DNAJC4*, and *MAPK8IP3*. “Butirosin and neomycin biosynthesis” and “Glyoxylate and dicarboxylate metabolism” were the most impacted pathways, underscoring their importance for functional adaptations of the mammary gland. In addition, *PLA2*, *CPT1*, *PLD*, *GGA*, *SRPRB*, and *AP4S1* were uncovered as node genes and participate in aspects of fatty acid metabolism. Our findings provide promising candidate genes for elucidating molecular mechanisms controlling mammary fatty acid metabolism during lactation in the dairy goat and should be explored further.

## Data Availability Statement

The sequencing data have been submitted to the NCBI SRA, and are accessible through the accession number PRJNA637690.

## Ethics Statement

The animal study was reviewed and approved by Animal Care and Use Committee of Northwest A&F University.

## Author Contributions

JL was responsible for the experimental design. JZ, WZ, HX, HW, and HS collected the tissue samples and isolated RNA for sequencing. JZ and HS performed the analysis of sequencing data and bioinformatics analysis. CL, JZ, HS, JL, and JJL wrote the manuscript. CL, JL, and JJL supervised the entire experiment, participated in result interpretation, and manuscript revision. All authors read and approved the final manuscript.

## Conflict of Interest

The authors declare that the research was conducted in the absence of any commercial or financial relationships that could be construed as a potential conflict of interest.

## Footnotes

1 http://www.bioinformatics.babraham.ac.uk/projects/fastqc/

## Supplementary Material

FIGURE S1 Analytical flowchart for digital gene expression sequencing in mammary gland of dairy goat. Nine mammary gland samples were collected in the stage of lactation, dry-off and non-lactation with three biological replicates per stage.

TABLE S1 The 15 most abundant genes involved in lipid transport and metabolism category (KOG database) in mammary gland.

TABLE S2 The 15 most abundant genes involved in intracellular trafficking, secretion, and vesicular transport category (KOG database) in mammary gland.

TABLE S3 The 15 most abundant genes involved in fatty acid metabolic process category (GO database) in mammary gland.

TABLE S4 The 15 most abundant genes involved in fatty acid biosynthetic process category (GO database) in mammary gland.

TABLE S5 Most abundant genes involved in fatty acid metabolism signaling pathway in mammary gland (KEGG database).

TABLE S6 Most abundant genes involved in fatty acid biosynthesis signaling pathway in mammary gland (KEGG database).

TABLE S7 DEGs involved in fatty acid metabolism among different stages of lactation (GO and KEGG databases).

TABLE S8 Most abundant DEGs involved in lipid metabolism among different stages of lactation (GO and KEGG databases).

TABLE S9 DEGs involved in protein metabolism among different stages of lactation (GO and KEGG databases).

TABLE S10 DEGs involved in lactose metabolism among different stages of lactation (GO and KEGG databases).

FILE S2 **(A)** List of all 51,299 defined unigenes. **(B)** List of all 9,131 differentially expressed genes (DEG). **(C)** List of all 246 enriched KEGG pathways. **(D)** List of specific genes from all 16 expression patterns through series-cluster analysis. **(E)** List of all DEGs for node genes selection through co-expression analysis.

FILE S3 Dynamic Impact Approach (DIA) analysis of differentially expressed genes during different stages of the lactation cycle. **(A)** General information during the process of DIA analysis using DEGs. **(B)** DEG data of the three comparison groups used in the DIA analysis. **(C)** Flux and impact generated by the DIA analysis. **(D)** Summary graph generated from the DIA analysis. **(E)** Overall pathway rank generated by the DIA analysis. **(F)** Pathway rank in each comparison generated by the DIA analysis.

## Abbreviations

ABCF2: ATP-binding cassette sub-family F member 2
ABDH2: acylglycerol lipase
ABI2: abl interactor 2
ACAA: acetyl-CoA acyltransferase
ACAA2: acetyl-CoA acyltransferase 2
ACADL: very long chain acyl-CoA dehydrogenase
ACADM: acyl-CoA dehydrogenase
ACADS: butyryl-CoA dehydrogenase
ACADSB: short/branched chain acyl-CoA dehydrogenase
ACAT1: acetyl-CoA C-acetyltransferase
ACBP: acyl-CoA-binding protein
ACOX1: acyl-CoA oxidase
ACSL: long-chain acyl-CoA synthetase
ADH: alcohol dehydrogenase
ADH1: alcohol dehydrogenase
ALDH: aldehyde dehydrogenase (NAD+)
ALDH1: aldehyde dehydrogenase (NAD+)
AP2M1: AP-2 complex subunit mu-1
AP4S1: AP-4 complex subunit sigma-1
ARF1: ADP-ribosylation factor 1
ARL5A: ADP-ribosylation factor-like 5A
B4GALT5: Beta-1,4-galactosyltransferase 5
BMP-3B: bone morphogenetic protein 3/3B
CD31: platelet/endothelial cell adhesion molecule
CHMP2A: charged multivesicular body protein 2A
CLTA, clathrin: light polypeptide A
COPS3: COP9 signalosome complex subunit 3
CPT1: choline-phosphate cytidylyltransferase
CPT1B: carnitine O-palmitoyltransferase 2
CPTA1: carnitine O-palmitoyltransferase 1
DEGs: differentially expressed genes
DEGS1: sphingolipid delta-4 desaturase
DGE: digital gene expression profiling
DIA: dynamic impact approach
DNAJC4: DnaJ homolog subfamily C member 4
EBP: cholestenol delta-isomerase
EHHADH: enoyl-CoA hydratase/3-hydroxyacyl-CoA dehydrogenase
EIF3S5: translation initiation factor eIF-3 subunit 5
ELOVL1: elongation of very long chain fatty acids protein 1
EPHA1: Eph receptor A1
FABP4: fatty acid-binding protein 4
FASN: fatty acid synthase
GCDH: glutaryl-CoA dehydrogenase
GGA: ADP-ribosylation factor-binding protein
GIT2: G protein-coupled receptor kinase interactor 2
GO: Gene Ontology
GPAT3: glycerol-3-phosphate O-acyltransferase 1/2
GPR64: G protein-coupled receptor 64
HLCS: acetyl-CoA carboxylase/biotin carboxylase
IGF1: insulin-like growth factor 1
IGFR2: Fc receptor IgG low affinity IIa
JNK, c-JunN-terminal kinase. KEGG: Kyoto Encyclopedia of Genes and Genomes
KLC: kinesin light chain
KOG: clusters of euKaryotic Orthologous Groups
LEF1: lymphoid enhancer-binding factor 1
lepB: signal peptidase I
MAPK8IP3: mitogen-activated protein kinase 8 interacting protein 3
MARCH2: E3 ubiquitin-protein ligase MARCH2
MARK: MAP/microtubule affinity-regulating kinase
MCAT: [acyl-carrier-protein] S-malonyltransferase
MDH2: malate dehydrogenase (NADP+)
MSMO1: methylsterol monooxygenase
MYB, Myb proto-oncogene protein;GJB2: gap junction protein beta 2
MYH: myosin heavy chain
MYSM1, protein Myb Like: SWIRM and MPN domains 1
NCAPH: condensin complex subunit 2
NDUFAB1: NADH dehydrogenase (ubiquinone) 1 alpha/beta subcomplex 1
NR1F1: nuclear receptor subfamily 1, group F member 1
OXSM: 3-oxoacyl-[acyl-carrier-protein] synthase II
PDP: pyruvate dehydrogenase phosphatase
PLA2: phospholipase A2
PLD: phospholipase D
PNK: polynucleotide kinase
POLG: DNA polymerase gamma 1
PPKAG1, 5’-AMP-activated protein kinase: regulatory gamma subunit
RAB11B: Ras-related protein Rab-11B
RAB1A: Ras-related protein Rab-1A
RNPEPL1: arginyl aminopeptidase-like 1
RUNX1T1: runt-related transcription factor 1 translocated to 1
SAPOSIN: saposin
SC5D: lathosterol oxidase
SCD: stearoyl-CoA desaturase
SDHD: succinate dehydrogenase (ubiquinone) membrane anchor subunit
SEC61: protein transport protein SEC61 subunit alpha
SEC61G: protein transport protein SEC61 subunit gamma and related proteins
SEMA6: semaphorin 6
SERPINE1: plasminogen activator inhibitor-1
SPAT1: spectrin alpha
SREBP: sterol-regulatory element binding proteins
SRPRB: signal recognition particle receptor subunit beta
SSR4: signal sequence receptor subunit 4
STAM: signal transducing adaptor molecule
STAT5A: signal transducer and activator of transcription 5A
STEM: Short Time-series Expression Miner
STK11: serine/threonine-protein kinase 11
TECR: enoyl reductase
TGF- β: growth factor β
TLL1: tolloid-like protein 1
USP16/29/37: ubiquitin carboxyl-terminal hydrolase 26/29/37.
