# Supplementary material for: Comprehensive Transcriptome Profiling of Dairy Goat Mammary Gland Identifies Genes and Networks Crucial for Lactation and Fatty Acid Metabolism
Source: Front Genet. 2020 Sep 25;11:878. doi: 10.3389/fgene.2020.00878 (PMC7545057; doi:10.3389/fgene.2020.00878)
Supplement: FIGURE S1 — Analytical flowchart for digital gene expression sequencing in mammary gland of dairy goat. Nine mammary gland samples were collected in the stage of lactation, dry-off and non-lactation with three biological replicates per stage. [file Table_1.doc]

**Comprehensive transcriptome profiling identifies crucial genes and networks in lactation and milk fatty acid metabolism of dairy goat**

Cong Li^1^†, Jiangjiang Zhu^1,2^†, Hengbo Shi^1,3^†, Jun Luo^1^*, Wangsheng Zhao^1^, Huaiping Shi^1^, Huifen Xu^1^, Hui Wang^1,2^, Juan. J. Loor^4^*


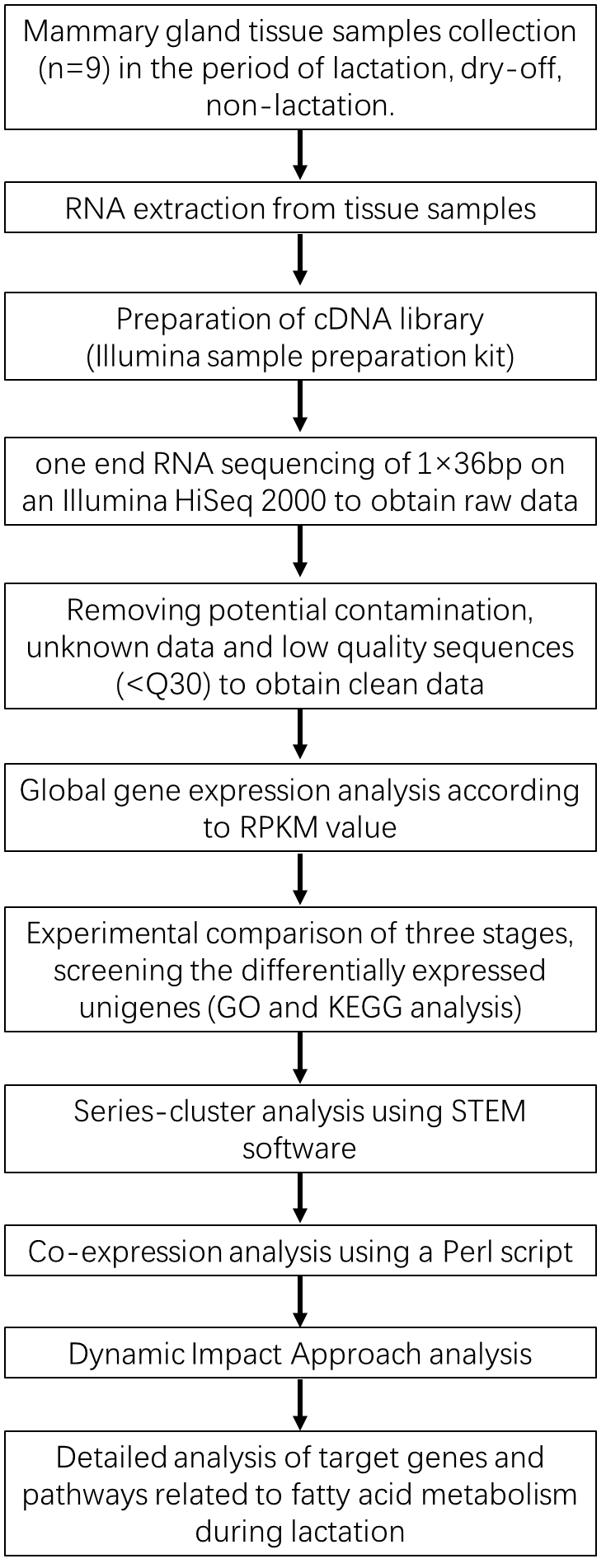
Supplementary Figure S1 Analytical flowchart for digital gene expression sequencing in mammary gland of dairy goat

Supplementary Table S1 The 15 most abundant genes involved in lipid transport and metabolism category (KOG database) in mammary gland

| **Number** | **Accession** | **Gene symbol** | **Length** | **Description** | **RPKM** | **Stage** |
| --- | --- | --- | --- | --- | --- | --- |
| 1 | comp11096_c1_seq1 | FABP3 | 2167 | fatty acid-binding protein 3, muscle and heart | 591.884 | L |
| 2 | comp44657_c0_seq1 | FASN | 8870 | fatty acid synthase, animal type | 376.322 | L |
| 3 | comp45341_c0_seq1 | SCD | 5401 | stearoyl-CoA desaturase (delta-9 desaturase) | 179.496 | L |
| 4 | comp27572_c0_seq1 | ACBP | 645 | diazepam-binding inhibitor (GABA receptor modulator, acyl-CoA-binding protein) | 120.752 | L |
| 5 | comp38982_c0_seq1 | SAPOSIN | 2700 | saposin | 109.100 | L |
| 6 | comp27637_c0_seq1 | FABP4 | 1305 | fatty acid-binding protein 4, adipocyte | 87.862 | L |
| 7 | comp45827_c0_seq1 | EBP | 6191 | cholestenol delta-isomerase | 80.036 | L |
| 8 | comp50561_c0_seq1 | PEMT | 886 | phosphatidylethanolamine N-methyltransferase | 77.726 | L |
| 9 | comp49204_c0_seq1 | RNPEPL1 | 2474 | arginyl aminopeptidase-like 1 | 59.140 | L |
| 10 | comp11140_c0_seq1 | TECR | 1200 | enoyl reductase | 46.990 | L |
| 11 | comp43091_c0_seq1 | AGPAT1 | 2766 | 1-acyl-sn-glycerol-3-phosphate acyltransferase | 32.966 | L |
| 12 | comp38568_c0_seq1 | DEGS1 | 1428 | sphingolipid delta-4 desaturase | 32.896 | L |
| 13 | comp39616_c0_seq1 | FABP5 | 1871 | fatty acid-binding protein 5, epidermal | 28.362 | L |
| 14 | comp35738_c0_seq1 | AACT | 1579 | acetyl-CoA C-acetyltransferase | 24.431 | L |
| 15 | comp33309_c0_seq1 | EHHADH | 1776 | 3-hydroxyacyl-CoA dehydrogenase | 24.134 | L |
| 1 | comp38982_c0_seq1 | SAPOSIN | 2700 | saposin | 453.244 | D |
| 2 | comp45827_c0_seq1 | EBP | 6191 | cholestenol delta-isomerase | 188.416 | D |
| 3 | comp44657_c0_seq1 | FASN | 8870 | fatty acid synthase, animal type | 120.146 | D |
| 4 | comp44190_c0_seq1 | PPT | 2231 | palmitoyl-protein thioesterase | 94.512 | D |
| 5 | comp27572_c0_seq1 | ACBP | 645 | diazepam-binding inhibitor (GABA receptor modulator, acyl-CoA-binding protein) | 86.386 | D |
| 6 | comp38568_c0_seq1 | DEGS1 | 1428 | sphingolipid delta-4 desaturase | 83.992 | D |
| 7 | comp35478_c0_seq1 | LPIN | 3293 | phosphatidate phosphatase | 78.883 | D |
| 8 | comp49204_c0_seq1 | RNPEPL1 | 2474 | arginyl aminopeptidase-like 1 | 76.903 | D |
| 9 | comp54985_c0_seq1 | NMT1 | 1814 | glycylpeptide N-tetradecanoyltransferase | 75.684 | D |
| 10 | comp35617_c0_seq1 | HADHA | 2748 | enoyl-CoA hydratase / long-chain 3-hydroxyacyl-CoA dehydrogenase | 71.864 | D |
| 11 | comp35754_c0_seq1 | LTA4H | 2128 | leukotriene-A4 hydrolase | 63.126 | D |
| 12 | comp27637_c0_seq1 | FABP4 | 1305 | fatty acid-binding protein 4, adipocyte | 55.813 | D |
| 13 | comp53980_c0_seq1 | NDUFAB1 | 659 | NADH dehydrogenase (ubiquinone) 1 alpha/beta subcomplex 1 | 52.232 | D |
| 14 | comp39616_c0_seq1 | FABP5 | 1871 | fatty acid-binding protein 5, epidermal | 49.517 | D |
| 15 | comp44246_c0_seq2 | LIPA | 2526 | lysosomal acid lipase/cholesteryl ester hydrolase | 46.147 | D |
| 1 | comp44657_c0_seq1 | FASN | 8870 | fatty acid synthase, animal type | 537.391 | NP |
| 2 | comp45341_c0_seq1 | SCD | 5401 | stearoyl-CoA desaturase (delta-9 desaturase) | 498.422 | NP |
| 3 | comp11096_c1_seq1 | FABP3 | 2167 | fatty acid-binding protein 3, muscle and heart | 366.782 | NP |
| 4 | comp38982_c0_seq1 | SAPOSIN | 2700 | saposin | 300.448 | NP |
| 5 | comp27572_c0_seq1 | ACBP | 645 | diazepam-binding inhibitor (GABA receptor modulator, acyl-CoA-binding protein) | 151.963 | NP |
| 6 | comp45827_c0_seq1 | EBP | 6191 | cholestenol delta-isomerase | 110.539 | NP |
| 7 | comp27637_c0_seq1 | FABP4 | 1305 | fatty acid-binding protein 4, adipocyte | 80.247 | NP |
| 8 | comp49204_c0_seq1 | RNPEPL1 | 2474 | arginyl aminopeptidase-like 1 | 74.896 | NP |
| 9 | comp11140_c0_seq1 | TECR | 1200 | enoyl reductase | 70.228 | NP |
| 10 | comp50561_c0_seq1 | PEMT | 886 | phosphatidylethanolamine N-methyltransferase | 65.912 | NP |
| 11 | comp35478_c0_seq1 | LPIN | 3293 | phosphatidate phosphatase | 54.200 | NP |
| 12 | comp46274_c1_seq7 | GPAM | 6799 | glycerol-3-phosphate O-acyltransferase 1/2 | 51.837 | NP |
| 13 | comp38568_c0_seq1 | DEGS1 | 1428 | sphingolipid delta-4 desaturase | 47.523 | NP |
| 14 | comp44190_c0_seq1 | PPT | 2231 | palmitoyl-protein thioesterase | 45.139 | NP |
| 15 | comp35754_c0_seq1 | LTA4H | 2128 | leukotriene-A4 hydrolase | 41.277 | NP |

Supplementary Table S2 The 15 most abundant genes involved in intracellular trafficking, secretion, and vesicular transport category (KOG database) in mammary gland

| **Number** | **Accession** | **Gene symbol** | **Length** | **Description** | **RPKM** | **Stage** |
| --- | --- | --- | --- | --- | --- | --- |
| 1 | comp11448_c0_seq1 | SSR4 | 789 | signal sequence receptor, delta (translocon-associated protein delta) | 451.609 | L |
| 2 | comp25213_c0_seq1 | lepB | 823 | signal peptidase I | 116.499 | L |
| 3 | comp11203_c0_seq1 | SEC61G | 587 | protein transport protein SEC61 subunit gamma and related proteins | 101.105 | L |
| 4 | comp44367_c0_seq1 | SEC61 | 3212 | protein transport protein SEC61 subunit alpha | 79.435 | L |
| 5 | comp38255_c0_seq1 | GASU_60350 | 2987 | fused signal recognition particle receptor | 79.226 | L |
| 6 | comp40682_c0_seq1 | AP2M1 | 1926 | AP-2 complex subunit mu-1 | 76.980 | L |
| 7 | comp24981_c0_seq1 | CLTA | 1055 | clathrin, light polypeptide A | 71.214 | L |
| 8 | comp34152_c0_seq1 | ARF1 | 1831 | ADP-ribosylation factor 1 | 69.620 | L |
| 9 | comp33648_c0_seq2 | RAB25 | 1095 | Ras-related protein Rab-25 | 63.801 | L |
| 10 | comp47598_c0_seq1 | CHMP2A | 913 | charged multivesicular body protein 2A | 52.602 | L |
| 11 | comp30951_c0_seq1 | RAB11B | 797 | Ras-related protein Rab-11B | 49.512 | L |
| 12 | comp25617_c0_seq1 | RAB1A | 2426 | Ras-related protein Rab-1A | 49.007 | L |
| 13 | comp35700_c0_seq1 | VAMP8 | 838 | vesicle-associated membrane protein 8 | 40.010 | L |
| 14 | comp21282_c0_seq1 | SDHD | 1336 | succinate dehydrogenase (ubiquinone) membrane anchor subunit | 35.818 | L |
| 15 | comp39679_c0_seq1 | ARF4 | 1733 | ADP-ribosylation factor 4 | 35.556 | L |
| 1 | comp11448_c0_seq1 | SSR4 | 789 | signal sequence receptor, delta (translocon-associated protein delta) | 311.538 | D |
| 2 | comp11203_c0_seq1 | SEC61G | 587 | protein transport protein SEC61 subunit gamma and related proteins | 189.764 | D |
| 3 | comp40682_c0_seq1 | AP2M1 | 1926 | AP-2 complex subunit mu-1 | 179.651 | D |
| 4 | comp34152_c0_seq1 | ARF1 | 1831 | ADP-ribosylation factor 1 | 155.150 | D |
| 5 | comp24981_c0_seq1 | CLTA | 1055 | clathrin, light polypeptide A | 128.831 | D |
| 6 | comp30951_c0_seq1 | RAB11B | 797 | Ras-related protein Rab-11B | 119.562 | D |
| 7 | comp47598_c0_seq1 | CHMP2A | 913 | charged multivesicular body protein 2A | 101.645 | D |
| 8 | comp20439_c0_seq1 | CHMP4 | 931 | charged multivesicular body protein 4 | 93.501 | D |
| 9 | comp25213_c0_seq1 | lepB | 823 | signal peptidase I | 92.320 | D |
| 10 | comp11788_c0_seq1 | RAB5C | 1519 | Ras-related protein Rab-5C | 88.367 | D |
| 11 | comp44367_c0_seq1 | SEC61 | 3212 | protein transport protein SEC61 subunit alpha | 87.199 | D |
| 12 | comp38871_c0_seq1 | COPA | 4308 | coatomer protein complex, subunit alpha (xenin) | 83.045 | D |
| 13 | comp25617_c0_seq1 | RAB1A | 2426 | Ras-related protein Rab-1A | 82.544 | D |
| 14 | comp21282_c0_seq1 | SDHD | 1336 | succinate dehydrogenase (ubiquinone) membrane anchor subunit | 82.023 | D |
| 15 | comp39679_c0_seq1 | ARF4 | 1733 | ADP-ribosylation factor 4 | 75.881 | D |
| 1 | comp11448_c0_seq1 | SSR4 | 789 | signal sequence receptor, delta (translocon-associated protein delta) | 414.403 | NP |
| 2 | comp40682_c0_seq1 | AP2M1 | 1926 | AP-2 complex subunit mu-1 | 148.348 | NP |
| 3 | comp11203_c0_seq1 | SEC61G | 587 | protein transport protein SEC61 subunit gamma and related proteins | 118.771 | NP |
| 4 | comp34152_c0_seq1 | ARF1 | 1831 | ADP-ribosylation factor 1 | 101.123 | NP |
| 5 | comp25213_c0_seq1 | lepB | 823 | signal peptidase I | 95.997 | NP |
| 6 | comp44367_c0_seq1 | SEC61 | 3212 | protein transport protein SEC61 subunit alpha | 91.840 | NP |
| 7 | comp24981_c0_seq1 | CLTA | 1055 | clathrin, light polypeptide A | 87.290 | NP |
| 8 | comp25617_c0_seq1 | RAB1A | 2426 | Ras-related protein Rab-1A | 83.3473 | NP |
| 9 | comp30951_c0_seq1 | RAB11B | 797 | Ras-related protein Rab-11B | 82.108 | NP |
| 10 | comp21282_c0_seq1 | SDHD | 1336 | succinate dehydrogenase (ubiquinone) membrane anchor subunit | 73.228 | NP |
| 11 | comp47598_c0_seq1 | CHMP2A | 913 | charged multivesicular body protein 2A | 59.647 | NP |
| 12 | comp38255_c0_seq1 | GASU_60350 | 2987 | fused signal recognition particle receptor | 58.369 | NP |
| 13 | comp38871_c0_seq1 | COPA | 4308 | coatomer protein complex, subunit alpha (xenin) | 52.810 | NP |
| 14 | comp11788_c0_seq1 | RAB5C | 1519 | Ras-related protein Rab-5C | 51.664 | NP |
| 15 | comp43804_c0_seq1 | YKT6 | 2436 | synaptobrevin homolog YKT6 | 51.191 | NP |

Supplementary Table S3 The 15 most abundant genes involved in fatty acid metabolic process category (GO database) in mammary gland

| **Number** | **Accession** | **Gene symbol** | **Length** | **Description** | **RPKM** | **Stage** |
| --- | --- | --- | --- | --- | --- | --- |
| 1 | comp33309_c0_seq1 | EHHADH | 1776 | enoyl-CoA hydratase / 3-hydroxyacyl-CoA dehydrogenase | 24.134 | L |
| 2 | comp35214_c0_seq2 | ACAA2 | 1568 | acetyl-CoA acyltransferase 2 | 13.984 | L |
| 3 | comp45232_c0_seq2 | ACSL | 3987 | long-chain acyl-CoA synthetase | 10.027 | L |
| 4 | comp37578_c0_seq1 | PLB1 | 2487 | lysophospholipase I | 6.800 | L |
| 5 | comp41821_c0_seq1 | ACAA | 2053 | acetyl-CoA acyltransferase | 6.309 | L |
| 6 | comp46062_c0_seq1 | SLC27A1 | 3248 | solute carrier family 27 (fatty acid transporter), member 1 | 5.924 | L |
| 7 | comp42079_c0_seq2 | ACADVL | 2221 | very long chain acyl-CoA dehydrogenase | 5.769 | L |
| 8 | comp40982_c0_seq1 | AACS | 1419 | acetoacetyl-CoA synthetase | 5.438 | L |
| 9 | comp15320_c0_seq1 | SLC27A5 | 857 | solute carrier family 27 (fatty acid transporter), member 5 | 5.242 | L |
| 10 | comp40009_c0_seq1 | ACADS | 1915 | butyryl-CoA dehydrogenase | 5.106 | L |
| 11 | comp39677_c0_seq5 | LYPLA2 | 1624 | lysophospholipase II | 5.080 | L |
| 12 | comp42079_c0_seq4 | ACADL | 2650 | very long chain acyl-CoA dehydrogenase | 4.857 | L |
| 13 | comp23651_c0_seq1 | ACADSB | 1946 | short/branched chain acyl-CoA dehydrogenase | 4.318 | L |
| 14 | comp21264_c0_seq1 | CPT1A | 831 | carnitine O-palmitoyltransferase 1 | 3.437 | L |
| 15 | comp20189_c0_seq1 | CPT1B | 2370 | carnitine O-palmitoyltransferase 2 | 3.377 | L |
| 1 | comp33309_c0_seq1 | EHHADH | 1776 | enoyl-CoA hydratase / 3-hydroxyacyl-CoA dehydrogenase | 34.567 | D |
| 2 | comp35214_c0_seq2 | ACAA2 | 1568 | acetyl-CoA acyltransferase 2 | 32.356 | D |
| 3 | comp41821_c0_seq1 | ACAA | 2053 | acetyl-CoA acyltransferase | 31.575 | D |
| 4 | comp33857_c0_seq2 | ACSL | 2403 | long-chain acyl-CoA synthetase | 29.374 | D |
| 5 | comp41377_c0_seq1 | CROT | 2722 | carnitine O-octanoyltransferase | 19.946 | D |
| 6 | comp45604_c0_seq1 | CPT1A | 4388 | carnitine O-palmitoyltransferase 1 | 17.528 | D |
| 7 | comp42079_c0_seq2 | ACADVL | 2221 | very long chain acyl-CoA dehydrogenase | 16.494 | D |
| 8 | comp20189_c0_seq1 | CPT1B | 2370 | carnitine O-palmitoyltransferase 2 | 15.472 | D |
| 9 | comp37578_c0_seq1 | PLB1 | 2487 | lysophospholipase I | 15.204 | D |
| 10 | comp40009_c0_seq1 | ACADS | 1915 | butyryl-CoA dehydrogenase | 14.073 | D |
| 11 | comp46062_c0_seq1 | SLC27A1 | 3248 | solute carrier family 27 (fatty acid transporter), member 1 | 12.949 | D |
| 12 | comp15320_c0_seq1 | SLC27A5 | 857 | solute carrier family 27 (fatty acid transporter), member 5 | 12.280 | D |
| 13 | comp39677_c0_seq5 | LYPLA2 | 1624 | lysophospholipase II | 11.776 | D |
| 14 | comp32015_c0_seq1 | ADIPOQ | 2534 | adiponectin | 8.288 | D |
| 15 | comp23651_c0_seq1 | ACADSB | 1946 | short/branched chain acyl-CoA dehydrogenase | 7.400 | D |
| 1 | comp33309_c0_seq1 | EHHADH | 1776 | enoyl-CoA hydratase / 3-hydroxyacyl-CoA dehydrogenase | 34.016 | NP |
| 2 | comp45232_c0_seq2 | ACSL | 3987 | long-chain acyl-CoA synthetase | 25.607 | NP |
| 3 | comp35214_c0_seq2 | ACAA2 | 1568 | acetyl-CoA acyltransferase 2 | 21.519 | NP |
| 4 | comp41821_c0_seq1 | ACAA | 2053 | acetyl-CoA acyltransferase | 18.322 | NP |
| 5 | comp37578_c0_seq1 | PLB1 | 2487 | lysophospholipase I | 12.673 | NP |
| 6 | comp40009_c0_seq1 | ACADS | 1915 | butyryl-CoA dehydrogenase | 12.148 | NP |
| 7 | comp46062_c0_seq1 | SLC27A1 | 3248 | solute carrier family 27 (fatty acid transporter), member 1 | 11.324 | NP |
| 8 | comp15320_c0_seq1 | SLC27A5 | 857 | solute carrier family 27 (fatty acid transporter), member 5 | 11.186 | NP |
| 9 | comp23651_c0_seq1 | ACADSB | 1946 | short/branched chain acyl-CoA dehydrogenase | 10.321 | NP |
| 10 | comp42079_c0_seq2 | ACADVL | 2221 | very long chain acyl-CoA dehydrogenase | 10.134 | NP |
| 11 | comp41008_c0_seq4 | AACS | 3112 | acetoacetyl-CoA synthetase | 10.001 | NP |
| 12 | comp45604_c0_seq1 | CPT1A | 4388 | carnitine O-palmitoyltransferase 1 | 9.265 | NP |
| 13 | comp20189_c0_seq1 | CPT1B | 2370 | carnitine O-palmitoyltransferase 2 | 8.930 | NP |
| 14 | comp39677_c0_seq5 | LYPLA2 | 1624 | lysophospholipase II | 8.824 | NP |
| 15 | comp41377_c0_seq1 | CROT | 2722 | carnitine O-octanoyltransferase | 5.360 | NP |

Supplementary Table S4 The 15 most abundant genes involved in fatty acid biosynthetic process category (GO database) in mammary gland

| **Number** | **Accession** | **Gene symbol** | **Length** | **Description** | **RPKM** | **Stage** |
| --- | --- | --- | --- | --- | --- | --- |
| 1 | comp44657_c0_seq1 | FASN | 8870 | fatty acid synthase, animal type | 376.322 | L |
| 2 | comp45341_c0_seq1 | SCD | 5401 | stearoyl-CoA desaturase (delta-9 desaturase) | 179.496 | L |
| 3 | comp11140_c0_seq1 | TECR | 1200 | enoyl reductase | 46.990 | L |
| 4 | comp45755_c0_seq2 | LPL | 7157 | lipoprotein lipase | 39.253 | L |
| 5 | comp38568_c0_seq1 | DEGS1 | 1428 | sphingolipid delta-4 desaturase | 32.896 | L |
| 6 | comp53980_c0_seq1 | NDUFAB1 | 659 | NADH dehydrogenase (ubiquinone) 1 alpha/beta subcomplex 1 | 20.657 | L |
| 7 | comp42936_c0_seq2 | MSMO1 | 1822 | methylsterol monooxygenase | 20.148 | L |
| 8 | comp38893_c0_seq2 | PRKAG1 | 1633 | 5'-AMP-activated protein kinase, regulatory gamma subunit | 15.357 | L |
| 9 | comp24175_c0_seq3 | ELOVL1 | 1711 | elongation of very long chain fatty acids protein 1 | 12.929 | L |
| 10 | comp22878_c0_seq1 | PRKAB1 | 2302 | 5'-AMP-activated protein kinase, regulatory beta subunit | 9.607 | L |
| 11 | comp40658_c0_seq1 | ABDH2 | 1521 | acylglycerol lipase | 9.438 | L |
| 12 | comp44482_c0_seq1 | SC5D | 2558 | lathosterol oxidase | 7.845 | L |
| 13 | comp42994_c0_seq2 | STK11 | 3110 | serine/threonine-protein kinase 11 | 7.423 | L |
| 14 | comp46074_c0_seq10 | HLCS | 8874 | acetyl-CoA carboxylase / biotin carboxylase | 7.075 | L |
| 15 | comp35037_c0_seq2 | HSD17B12 | 971 | estradiol 17beta-dehydrogenase | 5.369 | L |
| 1 | comp44657_c0_seq1 | FASN | 8870 | fatty acid synthase, animal type | 120.146 | D |
| 2 | comp38568_c0_seq1 | DEGS1 | 1428 | sphingolipid delta-4 desaturase | 83.992 | D |
| 3 | comp53980_c0_seq1 | NDUFAB1 | 659 | NADH dehydrogenase (ubiquinone) 1 alpha/beta subcomplex 1 | 52.232 | D |
| 4 | comp11140_c0_seq1 | TECR | 1200 | enoyl reductase | 41.992 | D |
| 5 | comp38458_c0_seq1 | CH25H | 1226 | cholesterol 25-hydroxylase | 28.651 | D |
| 6 | comp45341_c0_seq1 | SCD | 5401 | stearoyl-CoA desaturase (delta-9 desaturase) | 25.891 | D |
| 7 | comp38893_c0_seq2 | PRKAG1 | 1633 | 5'-AMP-activated protein kinase, regulatory gamma subunit | 22.484 | D |
| 8 | comp42936_c0_seq2 | MSMO1 | 1822 | methylsterol monooxygenase | 21.607 | D |
| 9 | comp24175_c0_seq3 | ELOVL1 | 1711 | elongation of very long chain fatty acids protein 1 | 21.448 | D |
| 10 | comp40658_c0_seq1 | ABDH2 | 1521 | acylglycerol lipase | 19.269 | D |
| 11 | comp42994_c0_seq2 | STK11 | 3110 | serine/threonine-protein kinase 11 | 15.131 | D |
| 12 | comp38579_c0_seq1 | ELOVL5 | 2037 | elongation of very long chain fatty acids protein 5 | 13.949 | D |
| 13 | comp9837_c0_seq1 | LXRA | 489 | nuclear receptor, subfamily 1, group H, member 3 | 12.440 | D |
| 14 | comp44482_c0_seq1 | SC5D | 2558 | lathosterol oxidase | 11.437 | D |
| 15 | comp91649_c0_seq1 | MECR | 1337 | mitochondrial trans-2-enoyl-CoA reductase | 11.333 | D |
| 1 | comp44657_c0_seq1 | FASN | 8870 | fatty acid synthase, animal type | 537.391 | NP |
| 2 | comp45341_c0_seq1 | SCD | 5401 | stearoyl-CoA desaturase (delta-9 desaturase) | 498.422 | NP |
| 3 | comp11140_c0_seq1 | TECR | 1200 | enoyl reductase | 70.228 | NP |
| 4 | comp38568_c0_seq1 | DEGS1 | 1428 | sphingolipid delta-4 desaturase | 47.523 | NP |
| 5 | comp42936_c0_seq2 | MSMO1 | 1822 | methylsterol monooxygenase | 28.351 | NP |
| 6 | comp53980_c0_seq1 | NDUFAB1 | 659 | NADH dehydrogenase (ubiquinone) 1 alpha/beta subcomplex 1 | 26.973 | NP |
| 7 | comp40658_c0_seq1 | ABDH2 | 1521 | acylglycerol lipase | 21.774 | NP |
| 8 | comp38893_c0_seq2 | PRKAG1 | 1633 | 5'-AMP-activated protein kinase, regulatory gamma subunit | 19.750 | NP |
| 9 | comp24175_c0_seq3 | ELOVL1 | 1711 | elongation of very long chain fatty acids protein 1 | 19.373 | NP |
| 10 | comp45755_c0_seq3 | LPL | 6957 | lipoprotein lipase | 16.495 | NP |
| 11 | comp44482_c0_seq1 | SC5D | 2558 | lathosterol oxidase | 13.375 | NP |
| 12 | comp41394_c0_seq1 | SCD | 2147 | stearoyl-CoA desaturase (delta-9 desaturase) | 13.307 | NP |
| 13 | comp42994_c0_seq2 | STK11 | 3110 | serine/threonine-protein kinase 11 | 12.521 | NP |
| 14 | comp46074_c0_seq10 | HLCS | 8874 | acetyl-CoA carboxylase / biotin carboxylase | 11.098 | NP |
| 15 | comp22878_c0_seq1 | PRKAB1 | 2302 | 5'-AMP-activated protein kinase, regulatory beta subunit | 9.427 | NP |

Supplementary Table S5 Most abundant genes involved in fatty acid metabolism signaling pathway in mammary gland (KEGG database)

| **Number** | **Accession** | **Gene symbol** | **Length** | **Description** | **RPKM** | **Stage** |
| --- | --- | --- | --- | --- | --- | --- |
| 1 | comp35738_c0_seq1 | ACAT1 | 1579 | acetyl-CoA C-acetyltransferase | 24.431 | L |
| 2 | comp33309_c0_seq1 | EHHADH | 1776 | enoyl-CoA hydratase / 3-hydroxyacyl-CoA dehydrogenase | 24.134 | L |
| 3 | comp35214_c0_seq2 | ACAA2 | 1568 | acetyl-CoA acyltransferase 2 | 13.984 | L |
| 4 | comp71400_c0_seq1 | ACADM | 2121 | acyl-CoA dehydrogenase | 13.118 | L |
| 5 | comp40861_c0_seq1 | ADH1 | 1693 | S-(hydroxymethyl) glutathione dehydrogenase / alcohol dehydrogenase | 12.093 | L |
| 6 | comp45232_c0_seq2 | ACSL | 3987 | long-chain acyl-CoA synthetase | 10.027 | L |
| 7 | comp39716_c0_seq1 | ALDH1 | 2019 | aldehyde dehydrogenase (NAD+) | 9.8382 | L |
| 8 | comp30379_c0_seq2 | GCDH | 1864 | glutaryl-CoA dehydrogenase | 8.8287 | L |
| 9 | comp42079_c0_seq2 | ACADL | 2221 | very long chain acyl-CoA dehydrogenase | 5.769 | L |
| 10 | comp40009_c0_seq1 | ACADS | 1915 | butyryl-CoA dehydrogenase | 5.106 | L |
| 11 | comp23651_c0_seq1 | ACADSB | 1946 | short/branched chain acyl-CoA dehydrogenase | 4.318 | L |
| 12 | comp41372_c0_seq1 | ACOX1 | 3219 | acyl-CoA oxidase | 3.660 | L |
| 13 | comp21264_c0_seq1 | CPT1A | 831 | carnitine O-palmitoyltransferase 1 | 3.437 | L |
| 14 | comp20189_c0_seq1 | CPT1B | 2370 | carnitine O-palmitoyltransferase 2 | 3.377 | L |
| 1 | comp39716_c0_seq1 | ALDH1 | 2019 | aldehyde dehydrogenase (NAD+) | 74.098 | NP |
| 2 | comp35617_c0_seq1 | EHHADH | 2748 | enoyl-CoA hydratase / long-chain 3-hydroxyacyl-CoA dehydrogenase | 71.864 | NP |
| 3 | comp35738_c0_seq1 | ACAT1 | 1579 | acetyl-CoA C-acetyltransferase | 32.677 | NP |
| 4 | comp35214_c0_seq2 | ACAA2 | 1568 | acetyl-CoA acyltransferase 2 | 32.356 | NP |
| 5 | comp33857_c0_seq2 | ACSL | 2403 | long-chain acyl-CoA synthetase | 29.374 | NP |
| 6 | comp40861_c0_seq1 | ADH1 | 1693 | S-(hydroxymethyl)glutathione dehydrogenase / alcohol dehydrogenase | 27.616 | NP |
| 7 | comp71400_c0_seq1 | ACADM | 2121 | acyl-CoA dehydrogenase | 27.581 | NP |
| 8 | comp45604_c0_seq1 | CPT1A | 4388 | carnitine O-palmitoyltransferase 1 | 17.528 | NP |
| 9 | comp43981_c0_seq1 | ACOX1 | 5965 | acyl-CoA oxidase | 17.277 | NP |
| 10 | comp42079_c0_seq2 | ACADL | 2221 | very long chain acyl-CoA dehydrogenase | 16.494 | NP |
| 11 | comp20189_c0_seq1 | CPT1B | 2370 | carnitine O-palmitoyltransferase 2 | 15.472 | NP |
| 12 | comp30379_c0_seq2 | GCDH | 1864 | glutaryl-CoA dehydrogenase | 14.748 | NP |
| 13 | comp40009_c0_seq1 | ACADS | 1915 | butyryl-CoA dehydrogenase | 14.073 | NP |
| 14 | comp23651_c0_seq1 | ACADSB | 1946 | short/branched chain acyl-CoA dehydrogenase | 7.400 | NP |
| 1 | comp39716_c0_seq1 | ALDH1 | 2019 | aldehyde dehydrogenase (NAD+) | 37.658 | D |
| 2 | comp33309_c0_seq1 | EHHADH | 1776 | enoyl-CoA hydratase / 3-hydroxyacyl-CoA dehydrogenase | 34.016 | D |
| 3 | comp45232_c0_seq2 | ACSL | 3987 | long-chain acyl-CoA synthetase | 25.607 | D |
| 4 | comp35214_c0_seq2 | ACAA2 | 1568 | acetyl-CoA acyltransferase 2 | 21.519 | D |
| 5 | comp35738_c0_seq1 | ACAT1 | 1579 | acetyl-CoA C-acetyltransferase | 20.994 | D |
| 6 | comp71400_c0_seq1 | ACADM | 2121 | acyl-CoA dehydrogenase | 18.479 | D |
| 7 | comp40861_c0_seq1 | ADH1 | 1693 | S-(hydroxymethyl)glutathione dehydrogenase / alcohol dehydrogenase | 18.222 | D |
| 8 | comp30379_c0_seq2 | GCDH | 1864 | glutaryl-CoA dehydrogenase | 15.089 | D |
| 9 | comp40009_c0_seq1 | ACADS | 1915 | butyryl-CoA dehydrogenase | 12.148 | D |
| 10 | comp43981_c0_seq1 | ACOX1 | 5965 | acyl-CoA oxidase | 11.626 | D |
| 11 | comp23651_c0_seq1 | ACADSB | 1946 | short/branched chain acyl-CoA dehydrogenase | 10.321 | D |
| 12 | comp42079_c0_seq2 | ACADL | 2221 | very long chain acyl-CoA dehydrogenase | 10.134 | D |
| 13 | comp45604_c0_seq1 | CPT1A | 4388 | carnitine O-palmitoyltransferase 1 | 9.265 | D |
| 14 | comp20189_c0_seq1 | CPT1B | 2370 | carnitine O-palmitoyltransferase 2 | 8.930 | D |

Supplementary Table S6 Most abundant genes involved in fatty acid biosynthesis signaling pathway in mammary gland (KEGG database)

| **Number** | **Accession** | **Gene symbol** | **Length** | **Description** | **RPKM** | **Stage** |
| --- | --- | --- | --- | --- | --- | --- |
| 1 | comp44657_c0_seq1 | FASN | 8870 | fatty acid synthase, animal type | 376.322 | L |
| 2 | comp46074_c0_seq10 | HLCS | 8874 | acetyl-CoA carboxylase / biotin carboxylase | 7.075 | L |
| 3 | comp21405_c0_seq1 | MCAT | 1820 | [acyl-carrier-protein] S-malonyltransferase | 2.367 | L |
| 4 | comp118602_c0_seq1 | OXSM | 1341 | 3-oxoacyl-[acyl-carrier-protein] synthase II | 1.768 | L |
| 1 | comp44657_c0_seq1 | FASN | 8870 | fatty acid synthase, animal type | 120.146 | D |
| 2 | comp118602_c0_seq1 | OXSM | 1341 | 3-oxoacyl-[acyl-carrier-protein] synthase II | 6.714 | D |
| 3 | comp21405_c0_seq1 | MCAT | 1820 | [acyl-carrier-protein] S-malonyltransferase | 6.604 | D |
| 4 | comp46074_c0_seq10 | HLCS | 8874 | acetyl-CoA carboxylase / biotin carboxylase | 3.938 | D |
| 1 | comp44657_c0_seq1 | FASN | 8870 | fatty acid synthase, animal type | 537.391 | NP |
| 2 | comp46074_c0_seq10 | HLCS | 8874 | acetyl-CoA carboxylase / biotin carboxylase | 11.098 | NP |
| 3 | comp118602_c0_seq1 | OXSM | 1341 | 3-oxoacyl-[acyl-carrier-protein] synthase II | 4.800 | NP |
| 4 | comp21405_c0_seq1 | MCAT | 1820 | [acyl-carrier-protein] S-malonyltransferase | 3.669 | NP |

Supplementary Table S7 DEG involved in fatty acid metabolism among different stages of lactation (GO and KEGG databases)

| **Number** | **GO/KEGG categories** | **DEGs symbol** | **DEGs description** | **Enrichment method** |
| --- | --- | --- | --- | --- |
| 1 | Fatty acid metabolic process | USP2_21 | ubiquitin carboxyl-terminal hydrolase 2/21 | GO/KEGG |
| 2 |  | BRD2 | bromodomain-containing protein 2 | GO/KEGG |
| 3 |  | DNAJC16 | DnaJ homolog, subfamily C, member 16 | GO/KEGG |
| 4 |  | ZSCAN2 | SCAN domain-containing zinc finger protein | GO/KEGG |
| 5 |  | ATXN3 | Ataxin-3 | GO/KEGG |
| 6 |  | HADHB | 3-hydroxyacyl-CoA dehydrogenase / 3a,7a,12a-trihydroxy-5b-cholest-24-enoyl-CoA hydratase | GO/KEGG |
| 7 |  | KCTD6 | BTB/POZ domain-containing protein 1/2 | GO/KEGG |
| 8 |  | ARL5A | ADP-ribosylation factor-like 5A | GO/KEGG |
| 9 |  | DTX | deltex | GO/KEGG |
| 10 |  | GZMH | granzyme H (cathepsin G-like 2) | GO/KEGG |
| 11 |  | CD22 | CD22 antigen | GO/KEGG |
| 12 |  | POLG | DNA polymerase gamma 1 | GO/KEGG |
| 13 |  | MYH | myosin heavy chain | GO/KEGG |
| 14 |  | NFATC | nuclear factor of activated T-cells, cytoplasmic, calcineurin-dependent | GO/KEGG |
| 15 |  | MARK | MAP/microtubule affinity-regulating kinase | GO/KEGG |
| 16 |  | STAM | signal transducing adaptor molecule | GO/KEGG |
| 17 |  | EPHA1 | Eph receptor A1 | GO/KEGG |
| 18 |  | TN | tenascin | GO/KEGG |
| 19 |  | ATP1B1 | F-type H+-transporting ATPase subunit beta | KEGG |
| 20 |  | ICMT | protein-S-isoprenylcysteine O-methyltransferase | KEGG |
| 21 |  | ETV3 | ETS translocation variant 3 | KEGG |
| 22 |  | ENPEP | glutamyl aminopeptidase | KEGG |
| 23 | Fatty acid metabolic process | PRODH | proline dehydrogenase | KEGG |
| 24 |  | NEUR | protein neuralized | KEGG |
| 25 |  | CBL | E3 ubiquitin-protein ligase CBL | KEGG |
| 26 |  | ADCY9 | adenylate cyclase 9 | KEGG |
| 27 |  | BCAM | Lutheran blood group glycoprotein | KEGG |
| 28 |  | SPSB3 | SPRY domain-containing SOCS box protein 3 | KEGG |
| 29 |  | LAG3 | lymphocyte-activation gene 3 | KEGG |
| 30 |  | ARHGDIA | Rho GDP-dissociation inhibitor | KEGG |
| 31 |  | APP | amyloid beta (A4) protein | KEGG |
| 32 |  | CHPF | chondroitin polymerizing factor 2 | KEGG |
| 33 |  | PCSK5 | proprotein convertase subtilisin/kexin type 5 | KEGG |
| 34 |  | URK1 | uridine kinase | KEGG |
| 35 |  | FOXO3 | forkhead box protein O3 | KEGG |
| 36 |  | CYCD3 | cyclin D3 | KEGG |
| 37 |  | HIST3H2A | histone H2A | GO |
| 38 |  | SYK | spleen tyrosine kinase | GO |
| 39 |  | CD34 | CD34 antigen | GO |
| 40 |  | CD58 | CD58 antigen | GO |
| 41 |  | EXOC7 | exocyst complex component 7 | GO |
| 42 |  | CALD1 | caldesmon | GO |
| 43 |  | ACD | adrenocortical dysplasia protein | GO |
| 44 |  | IGSF8 | immunoglobulin superfamily, member 8 | GO |
| 45 |  | PRMT2 | protein arginine N-methyltransferase 2 | GO |
| 46 |  | USP9_24 | ubiquitin carboxyl-terminal hydrolase 9/24 | GO |
| 47 |  | EVPL | envoplakin | GO |
| 48 | Fatty acid metabolic process | GSH-Px | glutathione peroxidase | GO |
| 49 | Fatty acid biosynthetic process | SEMA6 | semaphorin 6 | GO/KEGG |
| 50 |  | RUNX1T1 | runt-related transcription factor 1; translocated to, 1 | GO/KEGG |
| 51 |  | LEF1 | lymphoid enhancer-binding factor 1 | GO/KEGG |
| 52 |  | PC1 | polycystin 1 | KEGG |
| 53 |  | CDK | PCTAIRE protein kinase | GO |
| 54 |  | BPTF | nucleosome-remodeling factor subunit BPTF | GO |
| 55 |  | WNK | WNK lysine deficient protein kinase | GO |
| 56 |  | EIF2AK2 | eukaryotic translation initiation factor 2-alpha kinase | GO |
| 57 |  | SPSB3 | SPRY domain-containing SOCS box protein 3 | GO |
| 58 |  | URK1 | uridine kinase | GO |
| 59 |  | SDC1 | syndecan | GO |
| 60 |  | TUBG1 | tubulin gamma | GO |
| 61 |  | KZFP | KRAB domain-containing zinc finger protein | GO |
| 62 |  | ECA4 | Ca2+-transporting ATPase | GO |
| 63 |  | NFATC | nuclear factor of activated T-cells, cytoplasmic, calcineurin-dependent | GO |
| 64 |  | EXT1 | glucuronyl/N-acetylglucosaminyl transferase EXT1 | GO |
| 65 |  | CPXM1 | carboxypeptidase X1 | GO |
| 66 |  | TRIM3 | tripartite motif-containing protein 33 | GO |
| 67 |  | RPS4E | small subunit ribosomal protein S4e | GO |
| 68 |  | RAB27B | Ras-related protein Rab-27B | GO |
| 69 |  | ABCA6 | ATP-binding cassette, subfamily A (ABC1), member 6 | GO |
| 70 |  | ARHGDIA | Rho GDP-dissociation inhibitor | GO |
| 71 |  | PCGF5 | polycomb group RING finger protein 5 | GO |
| 72 |  | BCL10 | B-cell CLL/lymphoma 10 | GO |
| 73 | Fatty acid biosynthetic process | DTX | deltex | GO |
| 74 |  | HMGN3 | high-mobility group nucleosome-binding domain-containing protein 3 | GO |
| 75 |  | SIRT7 | NAD-dependent deacetylase sirtuin 7 | GO |
| 76 |  | INSP6 | inositol-hexakisphosphate kinase | GO |
| 77 |  | BOLA | major histocompatibility complex, class II | GO |
| 78 |  | IMPDH1 | IMP dehydrogenase | GO |
| 79 |  | HDAC7 | histone deacetylase 7 | GO |
| 80 |  | TFE3 | transcription factor E3-related | GO |
| 81 |  | LNPEP | cystinyl aminopeptidase | GO |
| 82 |  | TCF7L2 | transcription factor 7-like 2 | GO |
| 83 |  | XRCC3 | DNA-repair protein XRCC3 | GO |
| 84 |  | IGE1 | glycosyltransferase-like protein LARGE [CAZy:GT49] | GO |

Supplementary Table S8 Most abundant DEG involved in lipid metabolism among different stages of lactation (GO and KEGG databases)

| **Number** | **GO/KEGG categories** | **DEGs symbol** | **DEGs description** | **Enrichment method** |
| --- | --- | --- | --- | --- |
| 1 | Lipid metabolic process | UBE2L3 | ubiquitin-conjugating enzyme E2 L3 | GO/KEGG |
| 2 |  | CAPNS1 | calpain, small subunit 1 | KEGG |
| 3 |  | RPS4E | small subunit ribosomal protein S4e | KEGG |
| 4 |  | ARPC3 | actin related protein 2/3 complex, subunit 3 | KEGG |
| 5 |  | CDO | cysteine dioxygenase | KEGG |
| 6 |  | DYNLL1 | dynein light chain LC8-type | KEGG |
| 7 |  | RAB7A | Ras-related protein Rab-7A | KEGG |
| 8 |  | GPAT3 | glycerol-3-phosphate O-acyltransferase 1/2 | KEGG |
| 9 |  | ATP6V1E | V-type H+-transporting ATPase subunit E | KEGG |
| 10 |  | PRSS8 | protease, serine, 8 (prostasin) | KEGG |
| 11 |  | PSMCT1 | 26S proteasome regulatory subunit T1 | KEGG |
| 12 |  | GSK3A | glycogen synthase kinase 3 alpha | KEGG |
| 13 |  | DDA1 | DET1- and DDB1-associated protein 1 | KEGG |
| 14 |  | VDAC | voltage-dependent anion channel | KEGG |
| 15 |  | EHD2 | EH domain-containing protein 2 | KEGG |
| 16 |  | ASIP | agouti signaling protein | GO |
| 17 |  | AphC | peroxiredoxin (alkyl hydroperoxide reductase subunit C) | GO |
| 18 |  | MYL2 | myosin regulatory light chain 2 | GO |
| 19 |  | NKD | naked cuticle | GO |
| 20 |  | E2F5 | E2F transcription factor 4/5 | GO |
| 21 |  | MORF4L2 | mortality factor 4-like protein 2 | GO |
| 22 |  | ADH | S-(hydroxymethyl)glutathione dehydrogenase / alcohol dehydrogenase | GO |
| 23 |  | HO1 | heme oxygenase | GO |
| 24 | Lipid metabolic process | ALDH | aldehyde dehydrogenase (NAD+) | GO |
| 25 |  | PIGT | phosphatidylinositol glycan, class T | GO |
| 26 |  | NGLY1 | peptide-N4-(N-acetyl-beta-glucosaminyl)asparagine amidase | GO |
| 27 |  | CDIPT | CDP-diacylglycerol--inositol 3-phosphatidyltransferase | GO |
| 28 |  | MARCH2 | E3 ubiquitin-protein ligase MARCH2 | GO |
| 29 |  | MAP2K1 | mitogen-activated protein kinase kinase 1 | GO |
| 30 | Lipid biosynthetic process | ARPC1A | actin related protein 2/3 complex, subunit 1A/1B | KEGG |
| 31 |  | EIF3S5 | translation initiation factor eIF-3 subunit 5 | KEGG |
| 32 |  | ATP6V1G | V-type H+-transporting ATPase subunit G | KEGG |
| 33 |  | DCTN2 | dynactin 2 | KEGG |
| 34 |  | SPTB | spectrin beta | KEGG |
| 35 |  | PECAM1 | platelet/endothelial cell adhesion molecule | KEGG |
| 36 |  | SEMA4 | semaphorin 4 | KEGG |
| 37 |  | MLCK | myosin-light-chain kinase | KEGG |
| 38 |  | HDA6 | histone deacetylase 6/10 | KEGG |
| 39 |  | NR1F1 | nuclear receptor, subfamily 1, group F, member 1 | KEGG |
| 40 |  | Lin23 | F-box protein 23 | KEGG |
| 41 |  | ZKSCAN | KRAB and SCAN domains-containing zinc finger protein | KEGG |
| 42 |  | CLN3 | battenin | KEGG |
| 43 |  | HAT | histone acetyltransferase | KEGG |
| 44 |  | USP36 | ubiquitin carboxyl-terminal hydrolase 36/42 | KEGG |
| 45 |  | PSMCT1 | 26S proteasome regulatory subunit T1 | GO |
| 46 |  | MSN | moesin | GO |
| 47 |  | SLC17A5 | MFS transporter, ACS family, solute carrier family 17 (sodium-dependent inorganic phosphate cotransporter), member 5 | GO |
| 48 | Lipid biosynthetic process | GMNN | geminin | GO |
| 49 |  | DNAJC1 | DnaJ homolog, subfamily C, member 1 | GO |
| 50 |  | JARID1 | histone demethylase JARID1 | GO |
| 51 |  | MYH | myosin heavy chain | GO |
| 52 |  | HGS | hepatocyte growth factor-regulated tyrosine kinase substrate | GO |
| 53 |  | RBBP7 | histone-binding protein RBBP7 | GO |
| 54 |  | ITPK1 | inositol-1,3,4-trisphosphate 5/6-kinase | GO |
| 55 |  | TSP | thrombospondin | GO |
| 56 |  | ABCF2 | ATP-binding cassette, sub-family F, member 2 | GO |
| 57 |  | KCNJ8 | potassium inwardly-rectifying channel, subfamily J, member 8 | GO |
| 58 |  | RND1 | Rho family GTPase 1 | GO |
| 59 |  | DYRK | dual-specificity tyrosine-(Y)-phosphorylation regulated kinase | GO |

Supplementary Table S9 DEG involved in protein metabolism among different stages of lactation (GO and KEGG databases)

| **Number** | **GO/KEGG categories** | **DEGs symbol** | **DEGs description** | **Enrichment method** |
| --- | --- | --- | --- | --- |
| 1 | Protein export | YWHAZ | tyrosine 3-monooxygenase/tryptophan 5-monooxygenase activation protein | KEGG |
| 2 |  | GPAT3 | glycerol-3-phosphate O-acyltransferase 1/2 | KEGG |
| 3 |  | FNG | fringe | KEGG |
| 4 |  | CDC37 | cell division cycle protein 37 | KEGG |
| 5 |  | PSMA2 | 20S proteasome subunit alpha 2 | KEGG |
| 6 |  | APE1 | AP endonuclease 1 | KEGG |
| 7 |  | SHM2 | glycine hydroxymethyltransferase | KEGG |
| 8 |  | APBB1 | amyloid beta (A4) precursor protein-binding, family B, member 1 (Fe65) | KEGG |
| 9 |  | SLC16A1 | MFS transporter, MCP family, solute carrier family 16 (monocarboxylic acid transporters), member 1 | KEGG |
| 10 |  | TARS | threonyl-tRNA synthetase | KEGG |
| 11 |  | NR1F1 | nuclear receptor, subfamily 1, group F, member 1 | KEGG |
| 12 |  | NR2C1 | nuclear receptor, subfamily 2, group C, member 1 | KEGG |
| 13 |  | IGFR2 | Fc receptor, IgG, low affinity IIa | KEGG |
| 14 |  | MLKL | mixed lineage kinase domain-like | KEGG |
| 15 |  | SMARCAD1 | SWI/SNF-related matrix-associated actin-dependent regulator of chromatin subfamily A member 1 | KEGG |
| 16 | Protein metabolic process | TJP4 | tight junction protein 4 (peripheral) | GO |
| 17 |  | SNCAIP | synuclein, alpha interacting protein (synphilin) | GO |
| 18 |  | ZKSCAN | KRAB and SCAN domains-containing zinc finger protein | GO |
| 19 |  | MARCH2 | E3 ubiquitin-protein ligase MARCH2 | GO |
| 20 |  | USP36 | ubiquitin carboxyl-terminal hydrolase 36/42 | GO |
| 21 |  | NGLY1 | peptide-N4-(N-acetyl-beta-glucosaminyl) asparagine amidase | GO |
| 22 |  | HARS | histidyl-tRNA synthetase | GO |
| 23 |  | ADCY3 | adenylate cyclase 3 | GO |
| 24 |  | ADM | adrenomedullin | GO |
| 25 |  | PDP | pyruvate dehydrogenase phosphatase | GO |
| 26 |  | CARS | cysteinyl-tRNA synthetase | GO |
| 27 |  | MYH1 | myosin I | GO |
| 28 |  | FBXO6 | F-box protein 6 | GO |
| 29 |  | WDR20 | WD repeat-containing protein 22 | GO |
| 30 |  | FKBP9 | FK506-binding protein 9/10 | GO |
| 31 |  | HSF1 | heat shock transcription factor 1 | GO |
| 32 |  | IGF1 | insulin-like growth factor 1 | GO |
| 33 |  | IGE1 | glycosyltransferase-like protein LARGE [CAZy:GT49] | GO |
| 34 |  | STAT5A | signal transducer and activator of transcription 5A | GO |
| 35 |  | IGFR2 | Fc receptor, IgG, low affinity IIa | GO |
| 36 |  | DMPK | dystrophia myotonica-protein kinase | GO |
| 37 | Protein biosynthetic process | CHAF1B | chromatin assembly factor 1 subunit B | GO |
| 38 |  | QARS | glutamyl-tRNA synthetase | GO |
| 39 |  | CAPN7 | calpain-7 | GO |
| 40 |  | FBL6 | F-box and leucine-rich repeat protein 6 | GO |

Supplementary Table S10 DEG involved in lactose metabolism among different stages of lactation (GO and KEGG databases)

| **Number** | **GO/KEGG categories** | **DEGs symbol** | **DEGs description** | **Enrichment method** |
| --- | --- | --- | --- | --- |
| 1 | Lactose metabolic process | B4GALT5 | beta-1,4-galactosyltransferase 5 | GO/KEGG |
| 2 |  | ARRB | beta-arrestin | GO/KEGG |
| 3 |  | RAPGEF1 | Rap guanine nucleotide exchange factor (GEF) 1 | GO/KEGG |
| 4 |  | NAGA | alpha-N-acetylgalactosaminidase | KEGG |
| 5 |  | AHCY | adenosylhomocysteinase | KEGG |
| 6 |  | HSCB | molecular chaperone HscB | KEGG |
| 7 |  | MAP4K4 | mitogen-activated protein kinase kinase kinase kinase 4 | KEGG |
| 8 |  | JUNB | transcription factor jun-B | KEGG |
| 9 |  | MLCK | myosin-light-chain kinase | KEGG |
| 10 |  | IL6R | interleukin 6 receptor | KEGG |
| 11 |  | JMJD1 | jumonji domain-containing protein 1 | KEGG |
| 12 |  | FNG | fringe | KEGG |
| 13 |  | SIN3A | paired amphipathic helix protein Sin3a | KEGG |
| 14 |  | RNF138 | E3 ubiquitin-protein ligase RNF138 | KEGG |
| 15 |  | PAP | poly(A) polymerase | KEGG |
| 16 |  | SERPINE1 | plasminogen activator inhibitor-1 | GO |
| 17 |  | IKZF | IKAROS family zinc finger protein | GO |
| 18 |  | MDH2 | malate dehydrogenase (oxaloacetate-decarboxylating)(NADP+) | GO |
| 19 | Lactose biosynthetic process | BATF | ATF-like basic leucine zipper transcriptional factor | GO |
